# Ecdysonelactones, Ecdysteroids from the Tropical Eastern Pacific Zoantharian *Antipathozoanthus hickmani*

**DOI:** 10.3390/md16020058

**Published:** 2018-02-11

**Authors:** Paul O. Guillen, Kevin Calabro, Karla B. Jaramillo, Cristobal Dominguez, Grégory Genta-Jouve, Jenny Rodriguez, Olivier P. Thomas

**Affiliations:** 1 Escuela Superior Politécnica del Litoral, ESPOL, Centro Nacional de Acuacultura e Investigaciones Marinas, CENAIM, Campus Gustavo Galindo km. 30.5 vía Perimetral, Guayaquil P.O. Box 09-01-5863, Ecuador; P.GUILLENMENA1@nuigalway.ie (P.O.G.); K.JARAMILLOAGUILAR1@nuigalway.ie (K.B.J.); cdoming@espol.edu.ec (C.D.); 2 Marine Biodiscovery Laboratory, School of Chemistry and Ryan Institute, National University of Ireland, Galway (NUI Galway), University Road, H91 TK33 Galway, Ireland; kevin.calabro@nuigalway.ie; 3 School of Zoology and Ryan Institute, National University of Ireland Galway, University Road, H91 TK33 Galway, Ireland; 4 Laboratoire de Chimie-Toxicologie Analytique et Cellulaire (C-TAC) UMR CNRS 8638 COMETE, Université Paris Descartes, 4 Avenue De L’observatoire, 75006 Paris, France; gregory.genta-jouve@parisdescartes.fr

**Keywords:** ecdysteroids, ecdysonelactones, γ-lactone, Zoantharia, relative configuration, Cnidaria

## Abstract

Despite a large occurrence, especially over the Pacific Ocean, the chemical diversity of marine invertebrates belonging to the order Zoantharia is largely underexplored. For the two species of the genus *Antipathozoanthus* no chemical study has been reported so far. The first chemical investigation of *Antipathozoanthus hickmani* collected at the Marine Protected Area “El Pelado”, Santa Elena, Ecuador, led to the isolation of four new ecdysteroid derivatives named ecdysonelactones. The structures of ecdysonelactones A–D (**1**–**4**) were determined based on their spectroscopy data, including 1D and 2D NMR and HRMS. The four compounds of this family of ecdysteroids feature an unprecedented γ-lactone fused at the C-2/C-3 position of ring A. These derivatives exhibited neither antimicrobial nor cytotoxic activities.

## 1. Introduction

Zoantharians (Cnidaria:Anthozoa:Hexacorallia) are sessile marine invertebrates widely distributed in all oceans, and they can represent a high substrate cover in some shallow tropical coral reefs and deep sea environments [1,2]. While marine sponges have been deeply studied in the search for bioactive chemical entities, zoantharians are also known to biosynthesize a wide array of natural products with unique structural features and interesting bioactivity, such as zoanthamines [3,4,5]. Other families of natural products have also been isolated from different species of zoantharians, such as alkaloids [3,6,7,8] including zoanthoxanthins [9,10] and parazoanthines [11] isolated from the Mediterranean zoantharian *Parazoanthus axinellae*, prostaglandins such as PGA_2_ [12], fatty acids or palytoxin [13,14], one of the most toxic compounds, as well as ecdysteroids [15,16].

Studies on the diversity of marine invertebrates present off the Ecuadorian coast have shown that zoantharians are one of the most representative marine invertebrates inhabiting this maritime area [17,18,19]. The first records of zoantharian species in this maritime ecoregion were from the Galapagos Islands, where four new species and one new genus, *Terrazoanthus*, have been described. Within the context of a national project aiming at the description of the biological and chemical diversity of marine invertebrates from the marine protected area El Pelado of the Peninsula of Santa Elena, Ecuador, we noticed the presence of several species of zoantharians, representing a high substrate cover. The first inspection of the chemical diversity of one zoantharian of this ecoregion named *Terrazoanthus patagonichus* (formerly known as *T. onoi*) was very promising and revealed a new family of 2-aminoimidazole alkaloids named terrazoanthines [20]. A second species, commonly found in this area, was identified as *Antipathozoanthus hickmani*. The genus *Antipathozoanthus* belongs to the family Parazoanthidae and is composed by only two species so far: *A. macaronesicus* and *A. hickmani*, which are only found to be associated with black corals [21,22]. *Antipathozoanthus hickmani* was first recorded in the Tropical Eastern Pacific around the Galapagos Islands and later in Machalilla National Park, Ecuador, where this species was found as an epibiont of the antipatharian *Antiphates galapagensis* [17,19]. Interestingly, no studies on the chemical diversity of both species have been recorded so far, and we therefore decided to undertake the first chemical investigation of *A. hickmani*.

**Figure 1:**
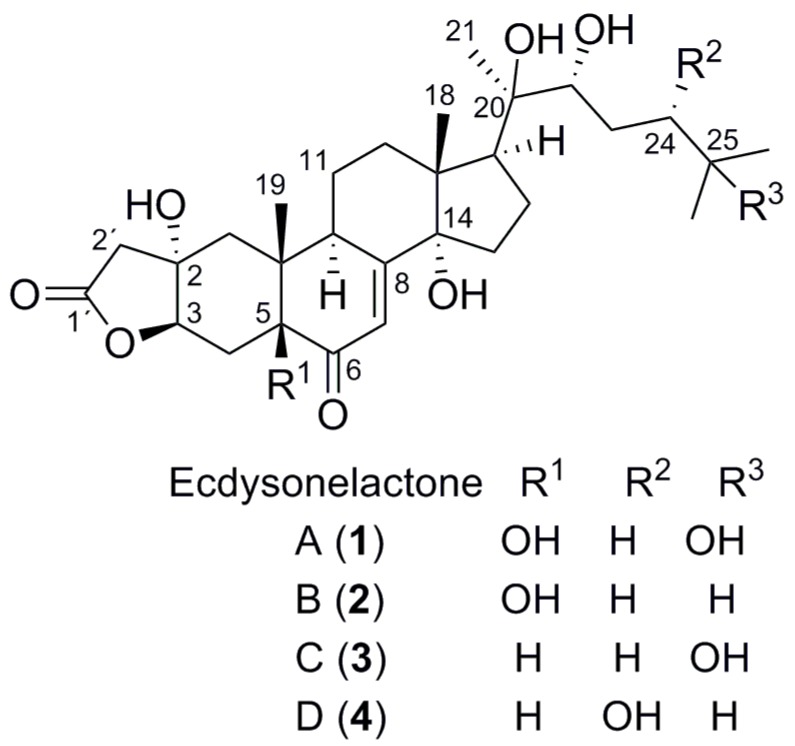
Structures of ecdysonelactones A–D (**1**–**4**) from *Antipathozoanthus hickmani.*

Herein, we report the isolation and structure elucidation of a new family of ecdysteroids named ecdysonelactones A–D (**1**–**4**), featuring for the first time a five-membered ring lactone moiety fused at the C-2 and C-3 positions of ring A (Figure 1). Three known compounds, including a lysine derivative first isolated from the marine sponge *Axinyssa terpnis* [23] and two ecdysteroids 20-hydroxyecdysone [16] and polypodine B [24,25], were also isolated from this zoantharian.

## 2. Results and Discussion

The frozen organism was first lyophilized, and the freeze-dried material was extracted with a mixture of solvents CH_3_OH/CH_2_Cl_2_ (*v*/*v*; 1:1) under sonication. The extract was first subjected to fractionation through reversed phase C18 Vacuum Liquid Chromatography (VLC) with solvents of decreasing polarity from H_2_O to CH_3_OH, until CH_2_Cl_2_ was reached. The methanolic fractions disclosed some interesting chemical profiles by UHPLC-DAD-ELSD and they were subjected to semipreparative Reversed Phase (RP) HPLC using a C18 column, yielding pure compounds ecdysonelactones A–D (**1**–**4**) along with the known compounds 20-hydroxyecdysone, polypodine B, and a lysine derivative.

Compound **1** was isolated as a white powder and (+)-HRESIMS analysis revealed a major peak at *m*/*z* 537.3070 [M + H]^+^, consistent with the molecular formula C_29_H_44_O_9_. The fragmentation pattern of the molecule indicated the loss of at least three molecules of water, suggesting the presence of three highly reactive alcohols in the molecule. NMR data were recorded in CD_3_OD even if most of the literature data are reported in C_5_D_5_N. Indeed, compounds were perfectly soluble in CD_3_OD with only rare overlaps of the signals with residual peaks of the solvents that did not prevent the structure elucidation of the compounds. The ^1^H NMR, ^13^C NMR, and HSQC analyses revealed one ester function, one α,β-unsaturated ketone, five oxygenated quaternary carbons, two oxygenated methines, three quaternary carbons, and five characteristic steroidal methyl signals. The ecdysteroid family was inferred from the signal at *δ*_H_ 5.88 (d, *J* = 2.5 Hz, 1H, H-7), corresponding to a trisubstituted olefinic proton. The H-7/C-5, C-9, and C-14 HMBC correlations confirmed the 7-en-6-one tetracyclic ring system and therefore the ecdysteroid family. Compared with other usual ecdysteroids found in zoantharians such as ecdysone, **1** lacked the oxymethine in position C-2, while the ^1^H NMR spectrum revealed a clear AB system at *δ*_H_ 2.74 (d, *J* = 17.0 Hz, H-2′a) and *δ*_H_ 2.56 (d, *J* = 17.0 Hz, H-2′b) (Table 1). The structure elucidation started with HMBC correlations from H_3_-19, allowing the assignment of the signals at C-1. Analysis of the HMBC spectrum revealed key H-2′/C-1, C-2, C-3 correlations showing that this methylene was directly linked to the A ring at C-2. The presence of a tertiary alcohol at C-2 was further proposed based on the signal at *δ*_C_ 74.2 (qC, C-2). Moreover, the HMBC spectrum revealed another key H-2′/C-1′ correlation with C-1′ corresponding to an ester function. Despite the lack of any other correlation with the latter carbonyl group, closure into a lactone at C-3 was suggested by the deshielding of the proton and carbon signals at the C-3 position of the A ring. This assumption was in accordance with the molecular formula of **1**. The presence of a hydroxy group at C-5 was then evidenced by HMBC correlations starting from H_3_-19, especially with another oxygenated quaternary carbon at *δ*_C_ 77.8 (qC, C-5). This oxidation pattern at C-5 is commonly found in ecdysteroids but more rarely in those isolated from zoantharians [26]. Focusing now on the side-chain of the steroid, the presence of a tertiary alcohol at C-20 and a secondary alcohol at C-22 was confirmed by key H_3_-21/C-22 and C-20 HMBC correlations. Finally, the presence of two terminal methyls and a tertiary alcohol at C-25 was demonstrated by additional key H_3_-26/C-24, C-25 and H_3_-27/C-24, C-25 HMBC correlations and a complete set of COSY correlations starting from H-22.

For the relative configuration of the side-chain, the ^1^H and ^13^C NMR chemical shifts of the signals from C-20 until C-27 for **1** were absolutely identical to those of 4-dehydroecdysterone reported by the group of Molinski, especially the signals and coupling constants [27]. For this compound, NMR data were also recorded in CD_3_OD, which allows a clean comparison, and the structure of the side-chain is therefore identical to the 17S*, 20R*, 22R* relative configurations for this part of the molecule. The *trans* C/D junction corresponding to 13R*, 14S* was also confirmed by the comparison of NMR signals with the same compound (Figure 1).

**Figure 2:**
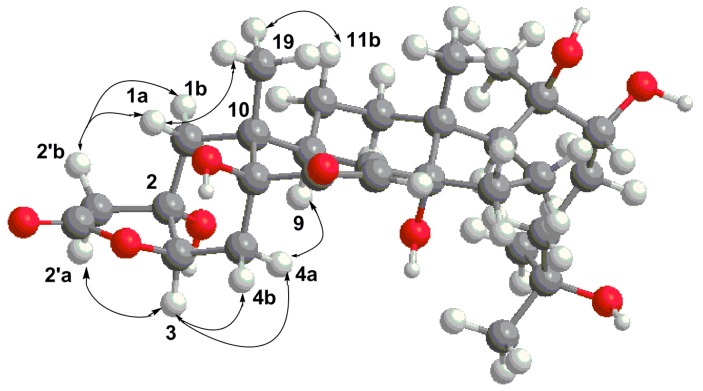
Minimum energy conformer for the *cis* epimer of **1**, where nOes are shown with arrows.

The relative configuration of the gamma lactone ring fused on the A ring then had to be assessed. First, the signal H-3 appeared as a doublet with ^3^*J*_H3-H4_ 4.5 Hz, which was only consistent with a chair conformation for ring A where the methyl H_3_-19 is placed in an equatorial position, like that already found for ecdysteroids of this type [28]. The oxygen at C-3 must then be *β* orientated and of the 3R*configuration. Indeed, for a α orientation of the oxygen at C-3, the proton H-3 would be placed in an axial position with a coupling constant of at least 10 Hz (See Supplementary Materials). For the relative configuration at the quaternary carbon C-2, we relied on the nOes between the different protons of the bicycle. The epimer with a *cis*-junction between the lactone and ring A was in accordance with the observed nOes and the distances measured (Figure 2), especially for the nOes H-9/H-4a, H_3_-19/H-1a, H-2′b/H-1a, and H-1b and H-3/H-2′a. All NMR data are therefore in agreement with this conformation of the 2S*, 3R*, 5S*, 9R*, 10R* epimer. Another confirmation came from the comparison between the chemical shifts of the four possible stereoisomers at C-2 and C-3 as well as literature data of non ecdysteroid analogues [29]. We did not investigate the absolute configuration of this molecule as, for all ecdysteroids described so far, all have the usual *β* orientation for the methyls H_3_-18 and H_3_-19 and therefore we propose the same absolute configuration as determined for all compounds of this large family.

Compound **2** was isolated as a white powder and its (+)-HRESIMS analysis suggested the molecular formula C_29_H_44_O_8_ with *m*/*z* 521.3104 [M + H]^+^. The fragmentation pattern evidenced the loss of three water molecules, and **2** was therefore thought to be a deoxygenated analogue of **1**. The ^13^C NMR spectrum of **2** indicated the presence of only six saturated and oxygenated carbons with a lack of one oxygenated quaternary carbon compared to **1**. The absence of the tertiary alcohol at C-25 was evidenced in the ^1^H NMR spectrum where the three singlets at *δ*_H_ 1.19 (6H, H_3_-21, H_3_-26) and 1.20 (3H, H_3_-27) in **1** were replaced by one remaining singlet at *δ*_H_ 1.19 (3H, H_3_-21) and two diastereotopic methyls with doublets at *δ*_H_ 0.90 (3H, H_3_-26) and 0.91 (3H, H_3_-27). The COSY spectrum confirmed the scalar coupling of these signals with a methine at C-25. Apart from these changes, all other signals in the ^1^H and ^13^C NMR spectra were like those of **1**, and we thus concluded that **2** is the C-25 deoxy analogue of **1**. The similarities between the ^1^H and ^13^C NMR data of **1** and **2** for C-20, C-21, and C-22 confirmed the same relative configuration at C-20 and C-22.

Compound **3** was isolated as a white powder and the molecular formula C_29_H_44_O_8_ was determined by (+)-HRESIMS with *m*/*z* 521.3107 [M + H]^+^. This compound is an isomer of **2**. The side-chain of **3** was found to be identical to that of **1**, as the same pattern was observed for the ^1^H NMR signals of the side-chain methyls. Therefore, and unlike **2**, **3** contains a tertiary alcohol at C-25. Because the molecular formula was found to be identical to that of **2**, we expected the loss of an oxygen in the polycyclic core. The absence of an oxygen was located at position C-5 due to the appearance of a new signal in the HSQC spectrum at *δ*_H_ 2.10 (m, 1H, H-5) and *δ*_C_ 52.1 (CH, C-5). Deoxygenation at this position is particularly common in ecdysteroids, especially in 20-hydroxyecdysone analogues already found in the zoantharian *Parazoanthus axinellae*, for example [30]. The location of this methine at C-5 was easily confirmed by a key H_3_-19/C-5 HMBC correlation. Due to the overlap of the ^1^H NMR signal H-5 with other signals, it was impossible to use nOe or coupling constant to assess the relative configuration at C-5. Gratifyingly, the presence of the same key H-4a/H-9 nOe as that found in **1** allowed us to conclude that the same *cis* junction existed between rings A and B. Indeed, this nOe would not be consistent with a *trans* decaline configuration between rings A and B. This assumption was further confirmed by very similar chemical shifts between the corresponding signals of **3** and those of analogues in the literature [27]. A *cis* junction between rings A and B seems to be a common feature of most ecdysteroids, an epimerization likely occurring because of the presence of the ketone at C-6. Identical chemical shifts in ^1^H and ^13^C NMR signals with those of **1** indicated the same relative configurations for the side-chain. Compound **3** is therefore the deoxy analogue of **1** at C-5.

Compound **4** was isolated as a white powder and its molecular formula C_29_H_45_O_8_ was determined by HRESIMS with *m*/*z* 521.3104 [M + H]^+^. This compound is therefore a third isomer of **2** and **3**. While the signals corresponding to the polycyclic part of the ecdysteroid were identical to those of **3**, major differences occurred for the signals of the side-chain. Indeed, like in **1** and **3**, the tertiary alcohol was not present at C-25 because the methyls H_3_-26 and H_3_-27 appeared as two diastereotopic methyls, as they did in **2**. The HSQC spectrum revealed changes for H-22 and another oxygenated methine overlapping with H-22 at *δ*_H_ 3.59 (m, 2H, H-22, and H-24). Based on key H_3_-21/C-22 and H_3_-26 and 27/C-24 HMBC correlations, we concluded that a hydroxylation occurred at C-24. To establish the relative configuration of the hydroxyl at C-24, we relied on literature data for pterosterone and its related epimer at C-24, even if those data were recorded in pyridine-*d*_5_ [28]. Comparison of the ^13^C NMR data of the signals corresponding to the side-chain of **1** recorded in CD_3_OD with those of abutasterone containing the same side-chain but recorded in C_5_D_5_N showed a maximum difference of 1.5 ppm [31]. While chemical shifts of *δ*_C_ 76.7 (C-22) and 77.5 (C-24) were observed in C_5_D_5_N for pterosterone with a 24*S* configuration, values of *δ*_C_ 73.4 (C-22) and 73.5 (C-24) were recorded for its epimer at C-24, 24-epipterosterone (24*R*) in the same solvent [28,32]. In the case of compound **4**, both carbons resonate at *δ*_C_ 77.6 (C-22) and 77.5 (C-24) in CD_3_OD, in total accordance with the values obtained for pterosterone. Because the change of solvent does not seem to strongly affect the chemical shifts of the ^13^C NMR signals, we assumed the same 24*S* configuration as pterosterone for compound **4**.

**Scheme 1:**
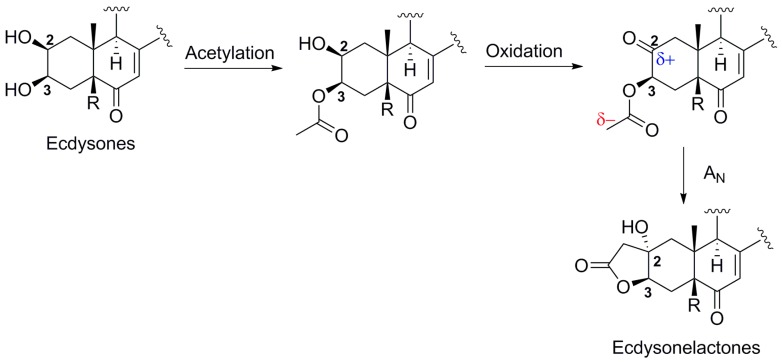
Proposed biosynthetic conversion of ecdysones into ecdysonelactones.

Ecdysteroids are known to be a widespread family of natural products produced by plants and arthropods, where they are known to play the role of molting hormones for arthropods, for example [33,34]. They are also widely found in some marine invertebrates and may be considered as chemomarkers of zoantharians, even if their ecological role is not known in this case. The first chemical investigation of a species of the genus *Antipathozoanthus* led to the discovery of a new family of ecdysteroids featuring an unprecedented *γ*-lactone ring fused between the positions C-2/C-3 of the A ring. The presence of a lactone ring in ecdysteroids has been found in compounds like cyasterone [35], ajugalactone [36], or even the very rare C-29 ecdysteroid named reptanslactone [37] with five- and six-membered ring lactones only present on the side chain. The formation of the five-membered ring lactone between the C-2 and C-3 positions of ring A in ecdysonelactones can be explained by the presence of very reactive secondary alcohols at C-2 and C-3 [38,39]. To explain the formation of the lactones, ecdysteroids from zoantharians seem to be prone to acetylation at position OH-2 or OH-3. Acetylation at OH-3 would lead to an acetylated product at this position that could undergo a cyclization into a *γ*-lactone on a ketone at C-2 (Scheme 1). Oxidation at positions C-2 or C-3 have already been observed [40], and such a nucleophilic addition of a methyl ester on an adjacent ketone was demonstrated synthetically in strongly basic conditions with a derivative of taxol [41]. An enzyme could catalyze the addition reaction in the case of this natural product and impose the observed stereochemistry. The presence of this enzyme seems restricted to the species *A. hickmani* among all studied zoantharians and other living organisms of the terrestrial world. Interestingly, only the *cis* fused product was formed through cyclization with the A ring, giving rise to the OH-2 on the *α* side of the polycyclic ring. 

Ecdysonelactones A–D (**1**–**4**) were tested for antibacterial activity against Gram-positive (Methicillin-resistant *Staphylococcus aureus* MRSA, *Staphylococcus aureus*) and Gram-negative bacteria (*Acinetobacter baumannii*, *Escherichia coli*, *Klebsiella pneumoniae*); antifungal activity against *Candida albicans*; and cytotoxicity against MCF-7 breast cancer, A2058 melanoma, HT-29 colon cancer, A549 lung cancer, and HepG2 hepatic cancer cell lines [42]. Despite the new lactone function fused on the A ring of the ecdysteroid, ecdysonelactones, like other ecdysteroids, did not exhibit significant antimicrobial or cytotoxic activities.

## 3. Materials and Methods

### 3.1. General Experimental Procedures

UV measurements were obtained by the extraction of the Diode Array Detector (DAD) signal of the Ultra-High Pressure Liquid Chromatography (UHPLC) Dionex Ultimate 3000 (Thermo Scientific, Waltham, MA, USA). Optical rotation measurements were obtained at the sodium D line (589.3 nm) with a 10-cm cell at 20 °C on a UniPol L1000 polarimeter (Schmidt + Haensch, Berlin, Germany). NMR spectra were acquired on an Agilent 600 MHz spectrometer equipped with a cryoprobe with pulse field gradient, and signals were referenced in ppm to the residual solvent signals (CD_3_OD, at *δ*_H_ 3.31 and *δ*_C_ 49.0). HRESIMS data were obtained with a UHR-qTOF Agilent 6540 mass spectrometer. Purification was carried out on a JASCO HPLC equipped with a PU4087 pump and a UV4070 UV/Vis detector.

### 3.2. Biological Material

The specimen identified as *Antipathozoanthus hickmani* (73 g dry weight) was collected by SCUBA at 24 m at the Marine Protected Area El Pelado (La Pared) of the Peninsula of Santa Elena, Ecuador. A voucher (150924EP01-02) of the sample is held at CENAIM-ESPOL (San Pedro, Santa Elena, Ecuador) (See Supplementary Materials). 

### 3.3. Extraction and Isolation

The freeze-dried sample (73.8 g) was extracted at room temperature with a mixture of solvents DCM/MeOH (1:1) three times (500 mL); each time with sonication and gravity filtration. The organic solvent was removed under pressure to obtain an extract (4.7 g). The extract was subjected to C18 reversed phase vacuum liquid chromatography (LiChroprep^®^ RP-18, 40–63 µm) using a mixture of solvents of decreasing polarity (1). H_2_O, (2). H_2_O/MeOH (1:1), (3). H_2_O/MeOH (1:3), (4). MeOH, (5). MeOH/DCM (3:1), (6). MeOH/DCM (1:1), and (7). DCM (using 500 mL of each solvent). Fractions 3 and 4 were purified by semi-preparative RP-HPLC with a C18 column (Restek, 10 × 250 mm, 5 µm), using the following elution steps with A CH_3_CN/acetic Acid 0.1%, B H_2_O/acetic acid 0.1% as mobile phases; isocratic for 0–20 min with A 22, B 78; linear gradient for 20–35 min until A 60, B 40; then isocratic for 35–45 min at a flow rate of 3.5 mL/min and UV detection at *λ* 254 nm for 50 min of acquisition time. These purifications yielded pure compounds; ecdysonelactone A (**1**, 0.9 mg), B (**2**, 1 mg), C (**3**, 0.7 mg), and D (**4**, 3 mg), and three known compounds; a lysine derivative (14.9 mg) [23], 20-hydroxyecdysone (1.3 mg) [16], and polypodine B (2.2 mg) [25].

### 3.4. Compound Characterization

#### 3.4.1. Ecdysonelactone A

**1**: White, amorphous solid; [*α*]${}_{D}^{20}$ +22 (*c* 0.1, CH_3_OH); UV (DAD) *λ*_max_ 248 nm; ^1^H NMR and ^13^C NMR data see Table 1; HRESIMS (+) *m*/*z* 537.3070 [M + H]^+^ (537.3058 calcd. for C_29_H_45_O_9_, *Δ* +2.2 ppm) and fragments 519.2963 [M + H–H_2_O]^+^ (519.2952 calcd. for C_29_H_43_O_8_); 501.2853 [M + H–2H_2_O]^+^ (501.2847 calcd. for C_29_H_41_O_7_); 483.2749 [M + H–3H_2_O]^+^ (483.2741 calcd. for C_29_H_39_O_6_).

#### 3.4.2. Ecdysonelactone B

**2**: White amorphous solid; [*α*]${}_{D}^{20}$ +24 (*c* 0.1, CH_3_OH); UV (DAD) *λ*_max_ 246 nm; ^1^H NMR and ^13^C NMR data see Table 1; HRESIMS (+) *m*/*z* 521.3104 [M + H–H_2_O]^+^ (521.3109 calcd. for C_29_H_45_O_8_ *Δ* −0.96 ppm) and fragments 503.3001 [M + H–2H_2_O]^+^ (503.3003 calcd. for C_29_H_43_O_7_); 485.2884 [M + H–3H_2_O]^+^ (485.2898 calcd. for C_29_H_41_O_6_); 467.2776 [M + H]^+^ (467.2792 calcd. for C_29_H_39_O_5_).

#### 3.4.3. Ecdysonelactone C

**3**: White, amorphous powder; [*α*]${}_{D}^{20}$ +30 (*c* 0.1, CH_3_OH); UV (DAD) *λ*_max_ 248 nm; ^1^H NMR and ^13^C NMR data see Table 1; HRESIMS (+) *m*/*z* [M + H–H_2_O]^+^ 521.3107 (521.3109 calcd. for C_29_H_45_O_8_ *Δ* −0.38 ppm) and fragments 503.3006 [M + H–2H_2_O]^+^ (503.3003 calcd. for C_29_H_43_O_7_); 485.2894 [M + H–3H_2_O]^+^ (485.2898 calcd. for C_29_H_41_O_6_); 467.2787 [M + H]^+^ (467.2792 calcd. for C_29_H_39_O_5_).

#### 3.4.4. Ecdysonelactone D

**4**: White, amorphous powder; [*α*]${}_{D}^{20}$ +29 (*c* 0.1, CH_3_OH); UV (DAD) *λ*_max_ 240 nm; ^1^H NMR and ^13^C NMR data, see Table 1; HRESIMS (+) *m*/*z* 521.3104 [M + H–H_2_O]^+^ (521.3109 calcd. for C_29_H_45_O_8_ *Δ* –0.96 ppm) and fragments 503.2993 [M + H–2H_2_O]^+^ (503.3003 calcd. for C_29_H_43_O_7_); 485.2885 [M + H–3H_2_O]^+^ (485.2898 calcd. for C_29_H_41_O_6_); 467.2868 [M + H]^+^ (467.2792 calcd. for C_29_H_39_O_5_).

### 3.5. Computational Analyses

The most stable conformer for the four diastereoisomers of **1** were generated using the MMFF94 force field (MarvinView, version 6.2.2, calculation module developed by ChemAxon, Budapest, Hungary) and the geometry described in Ohta et al. as the initial input [28]. The ^3^*J*_H,H_ couplings were predicted using MSpin (Mestrelab Research, Santiago de Compostela, Spain) for each diasteroisomer.

## 4. Conclusions

Ecdysonelactones A–D (**1**–**4**), isolated from the Tropical Eastern Pacific zoantharian *A. hickmani*, are new members of the ecdysteroid family of natural products as they feature an unprecedented *γ*-lactone fused at C-2 and C-3 of ring A. To our knowledge, this is the first report of ecdysteroids containing a five-membered lactone fused to ring A. The fusion of the lactone moiety to ring A could originate from a cyclization of the 3-*O* acetate onto a ketone at C-2.

## Figures and Tables

**Table 1:** NMR spectroscopic data for ecdysonelactones A–D (**1**–**4**) in CD_3_OD (600 MHz for ^1^H NMR data and 150 MHz for ^13^C NMR data).

No.	1		2		3		4	
	*δ*_H_, Mult. (*J* in Hz)	*δ*_C_	*δ*_H_, Mult. (*J* in Hz)	*δ*_C_	*δ*_H_, Mult. (*J* in Hz)	*δ*_C_	*δ*_H_, Mult. (*J* in Hz)	*δ*_C_
**1a** **1b**	1.87, d (15.0)	38.4	1.85, d (15.0)	38.4	2.11, m	41.9	2.13, m	41.9
	1.71, d (15.0)		1.70, d (15.0)		1.09, d (15.0)		1.10, d (15.0)	
**2**	-	74.2	-	74.2	-	74.0	-	74.0
**3**	4.50, br d (4.5)	82.5	4.49, br d (4.5)	82.5	4.40, br s	82.0	4.40, br s	82.1
**4a** **4b**	2.35, dd (16.0, 4.5)	31.9	2.35, m	31.9	2.13, m	25.1	2.11, m	25.8
	1.91, m		1.92, m		1.93, m		1.93, m	
**5**	-	77.8	-	77.8	2.10, m	52.1	2.11, m	52.1
**6**	-	203.9	-	203.9	-	205.3	-	203.7
**7**	5.88, d (2.5)	120.1	5.86, d (2.5)	120.1	5.83, d (2.0)	121.6	5.83, d (2.0)	121.6
**8**	-	169.2		169.2	-	169.3	-	169.2
**9**	3.91, m	39.2	3.90, m	39.2	3.80, ddd (11.5, 6.5, 2.0)	35.7	3.80, br dd (11.5, 6.5)	35.7
**10**	-	42.4	-	42.4	-	37.0	-	37.0
**11a** **11b**	1.93, m	22.3	1.92, m	22.3	1.93, m	21.4	1.93, m	21.4
	1.68, m		1.68, m		1.65, m		1.64, m	
**12a** **12b**	2.14, td (13.0, 4.5)	32.5	2.12, td (13.0, 4.5)	32.6	2.15, m	32.5	2.14, m	32.5
	1.83, td (13.0, 4.5)		1.81, m		1.84, m		1.84, dd (13.5, 3.5)	
**13**	-	48.7	-	48.6	-	48.8	-	48.7
**14**	-	84.9	-	84.9	-	85.1	-	85.0
**15a** **15b**	1.97	31.9	1.94, m	31.9	1.98, m	31.9	1.93, m	31.9
	1.59, t (10.0)		1.59, m		1.59, t (10.0)		1.61, m	
**16a** **16b**	1.98, m	21.5	1.97, m	21.5	1.98, m	21.5	1.99, m	21.5
	1.73, m		1.69, m		1.73, m		1.71, m	
**17**	2.39, m	50.4	2.35, m	50.4	2.39, t (8.6)	50.5	2.34, m	50.4
**18**	0.90, s	18.1	0.88, s	18.1	0.89, s	18.1	0.89, s	18.1
**19**	0.86, s	16.7	0.85, s	16.7	0.93, s	24.1	0.93, s	24.1
**20**	-	77.9	-	77.9	-	77.9	-	77.8
**21**	1.19, s	21.0	1.16, s	21.0	1.19, s	21.0	1.21, s	20.9
**22**	3.32, m	78.4	3.33, m	78.0	3.33, m	78.4	3.59, m	77.6
**23a** **23b**	1.67, m	27.3	1.56, m	30.5	1.66, m	27.3	1.71, m	35.7
	1.28, m		1.21, m		1.28, m		1.34, m	
**24a** **24b**	1.80, m	42.4	1.46, m	37.7	1.80, td (12.5, 4.5)	42.4	3.59, m	77.5
	1.42, td (12.5, 4.5)		1.22, m		1.43, td (12.5, 4.5)			
**25**		71.3	1.56, m	29.2		71.3	1.69, m	34.1
**26**	1.19, s	28.9	0.91, d (6.5)	23.4	1.19, s	28.9	0.95, d (7.0)	19.4
**27**	1.20, s	29.7	0.90, d (6.5)	22.8	1.20, s	29.7	0.91, d (7.0)	17.0
**1′**		176.3		176.2		176.1		176.0
**2′a** **2′b**	2.74, d (17.0)	47.5	2.72, d (17.0)	47.5	2.71, d (16.5)	47.1	2.70, d (16.5)	47.4
	2.56, d (17.0)		2.56, d (17.0)		2.50, d (16.5)		2.49, d (16.5)	

## Supplementary Materials

Supplementary materials, including taxonomic data, HRMS, 1D and 2D NMR spectra for compounds **1**–**4**, and computational details for **1**, are available on-line at http://www.mdpi.com/1660-3397/16/2/58/s1.
