# Supplementary material for: Ecdysonelactones, Ecdysteroids from the Tropical Eastern Pacific Zoantharian Antipathozoanthus hickmani
Source: Mar Drugs. 2018 Feb 11;16(2):58. doi: 10.3390/md16020058 (PMC5852486; doi:10.3390/md16020058)
Supplement: Supplementary file 1 [file marinedrugs-16-00058-s001.pdf]

Supplementary Information for

Ecdysonelactones, ecdysteroids from the Tropical Eastern Pacific Zoantharian *Antipathozoanthus hickmani*

Paul O. Guillen [1,2], Kevin Calabro [2], Karla B. Jaramillo [1,3], Cristobal Dominguez [1], Grégory Genta-Jouve [4], Jenny Rodriguez [1,*] and Olivier P. Thomas [2,*]

1 Escuela Superior Politécnica del Litoral, ESPOL, Centro Nacional de Acuacultura e Investigaciones Marinas, CENAIM, Campus Gustavo Galindo km. 30.5 vía Perimetral, P.O. Box 09-01-5863, Guayaquil Ecuador; P.GUILLENMENA1@nuigalway.ie (P.O.G.); K.JARAMILLOAGUILAR1@nuigalway.ie (K.B.J.); cdoming@espol.edu.ec (C.D.)

2 Marine Biodiscovery Laboratory, School of Chemistry and Ryan Institute, National University of Ireland, Galway (NUI Galway), University Road, H91 TK33 Galway, Ireland; kevin.calabro@nuigalway.ie (KC)

3 School of Zoology and Ryan Institute, National University of Ireland Galway, University Road, H91 TK33 Galway, Ireland

4 Laboratoire de Chimie-Toxicologie Analytique et Cellulaire (C-TAC) UMR CNRS 8638 COMETE, Université Paris Descartes, 4 avenue de l'observatoire, 75006 Paris, France; gregory.genta-jouve@parisdescartes.fr

* Correspondance: jenrodri@espol.edu.ec (J.R.); olivier.thomas@nuigalway.ie Tel.: ++353 (0)91493563 (O.P.T.)

P2 **Taxonomic identification of the specimen**
P5 **Figure S1**. (+)-HRESIMS analysis of **1**
P6 **Figure S2**. ^{1}H NMR spectrum of **1** at 600 MHz in CD_3OD
P7 **Figure S3**. COSY NMR spectrum of **1** at 600 MHz in CD_3OD
P8 **Figure S4**. ^{13}C NMR spectrum of **1** at 150 MHz in CD_3OD
P9 **Figure S5**. HSQC NMR spectrum of **1** at 600MHz in CD_3OD
P10 **Figure S6**. HMBC NMR spectrum of **1** at 600MHz in CD_3OD
P11 **Figure S7**. NOESY NMR spectrum of **1** at 600MHz in CD_3OD
P12 **Figure S8**. UHPLC-qToF analysis of **2** in (+)-ESI
P13 **Figure S9**. ^{1}H NMR spectrum of **2** at 600 MHz in CD_3OD
P14 **Figure S10**. COSY NMR spectrum of **2** at 600 MHz in CD_3OD
P15 **Figure S11**. ^{13}C NMR spectrum of **2** at 150 MHz in CD_3OD
P16 **Figure S12**. HSQC NMR spectrum of **2** at 600 MHz in CD_3OD
P17 **Figure S13**. HMBC NMR spectrum of **2** at 600 MHz in CD_3OD
P18 **Figure S14**. UHPLC-qToF analysis of **3** in (+)-ESI
P19 **Figure S15**. ^{1}H NMR spectrum of **3** at 600 MHz in CD_3OD
P20 **Figure S16**. COSY NMR spectrum of **3** at 600 MHz in CD_3OD
P21 **Figure S17**. ^{13}C NMR spectrum of **3** at 150 MHz in CD_3OD
P22 **Figure S18**. HSQC NMR spectrum of **3** at 600 MHz in CD_3OD
P23 **Figure S19**. HMBC NMR spectrum of **3** at 600 MHz in CD_3OD
P24 **Figure S20**. NOESY NMR spectrum of **3** at 600 MHz in CD_3OD
P25 **Figure S21**. UHPLC-qToF analysis of **4** in (+)-ESI
P26 **Figure S22**. ^{1}H NMR spectrum of **4** at 600 MHz in CD3OD
P27 **Figure S23**. COSY NMR spectrum of **4** at 600 MHz in CD_3OD
P28 **Figure S24**. ^{13}C NMR spectrum of **4** at 150 MHz in CD_3OD
P29 **Figure S25**. HSQC NMR spectrum of **4** at 600 MHz in CD_3OD
P30 **Figure S26**. HMBC NMR spectrum of **4** at 600 MHz in CD_3OD
P31 **Computational details**

Taxonomic identification of the specimen

Biological material. *Antipathozoanthus hickmani* is a Macrocnemic zoantharian of the family of Parazoanthidae (Cnidaria, Anthozoa, Hexacorallia, Zoantharia, Macrocnemina).[1] It was identified for the first time in the Pacific Ocean at the Galapagos Islands and then it was reported in the northern coast of mainland Ecuador.[2] The colony sampled had approximately 124 polyps, they were collected at 24 m depth from the branches of the black coral *Myriopathes panamensis* at the Marine Protected Area "El Pelado" Ecuador (See Figure below). Most of them have been found growing on the branches of two black corals *Antipathes galapagensis* and *Myriopathes* spp.

Sampling. The specimen was collected by SCUBA diving in September 2015 at 24 m depth from the site named "La Pared" (1°55′58.24′′S/ 80°47′32.83′′W) at the rocky reefs of the Marine Protected Area El Pelado. (Site type: vertical cliff of 31 m depth), East coast of mainland Ecuador. Interestingly, the specimens observed of *A. hickmani* always were found in the deepest locations from the MPA (2 of 13 different sites). The separation from the branches of the black corals was performed cautiously to avoid any contamination from the host tissues. The specimen was fixed partly in 4% formaldehyde and 95% ethanol for systematic analyses and the remaining material was kept in -80 °C freezer for chemical compound analyses. A voucher sample is kept at CENAIM-ESPOL under the number 150924EP01-02.

Systematic analysis

Morphological characterization. The morphological characters were analysed from the formalin sample, in situ annotations and *in situ* pictures to determine the polyp size (height, width, and oral disk diameter), number of mesenteries, number of tentacles, type of sphincter, colours (tentacles, oral disk, column and coenenchyme), sand encrustations and related/substrate type.

DNA extraction and sequencing. The DNA data were extracted from ethanol 95% fixed samples. DNA extraction was obtained according to the guanidine extraction protocol as previously described.[3] Samples were them amplified for mitochondrial 16S rDNA using the primers 16Sant0a,16SbmoH based in the thermal cycle conditions mentioned.[4] For the nuclear internal transcribed spacer region (ITS-2) rDNA using the primers Zoan-f, Zoan-r and the protocol described with standard Taq polymerase.[5] The amplified DNA products were sent to the

---

[1] Reimer, J. D. & Fujii, T. Four new species and one new genus of zoanthids (Cnidaria, Hexacorallia) from the Galápagos Islands. ZooKeys 42, 1, (2010).

[2] Bo, M. et al. Black coral assemblages from Machalilla National Park (Ecuador). Pacific Science 66, 63-81, (2012).

[3] Sinniger, F., Reimer, J. D. & Pawlowski, J. The Parazoanthidae (Hexacorallia: Zoantharia) DNA taxonomy: description of two new genera. Marine Biodiversity 40, 57-70, (2010).

[4] Sinniger, F., Montoya-Burgos, J.-I., Chevaldonné, P. & Pawlowski, J. Phylogeny of the order Zoantharia (Anthozoa, Hexacorallia) based on the mitochondrial ribosomal genes. Marine Biology 147, 1121-1128, (2005).

[5] Reimer, J. D., Sinniger, F., Fujiwara, Y., Hirano, S. & Maruyama, T. Morphological and molecular characterization of Abyssoanthus nankaiensis,, a new family, new genus and new species of deep-sea zoanthid (Anthozoa : Hexacorallia : Zoantharia) from a north-west Pacific methane cold seep. Invertebrate Systematics 21, 255-262, (2007).

sequencing company (MacroGen Inc, Korea). The *A. hickmani* sequences obtained of were deposited in GenBank, with the accessions numbers XXXXXXXX and XXXXXXX.

Phylogenetic analysis. The resulting sequences were manually assembled, and the chromatograms were checked for quality using Geneious 10.2.2. The *A. hickmani* sequences were compared with publicly available sequences from the original *A. hickmani* and other zoantharians using the National Centre for Biotechnology Information's Basic Local Alignment Search Tool (NCBI BLAST).

RESULTS

Systematics.

Antipathozoanthus hickmani **(Reimer & Fujii, 2010)**

Material examined. Specimen number: 150924EP01-02. Type locality: East coast of mainland Ecuador, Santa Elena province, and Marine Protected Area "El Pelado" site "Bajo La Pared", geographical coordinates: 1°55'58.24''S/ 80°47'32.83''W.

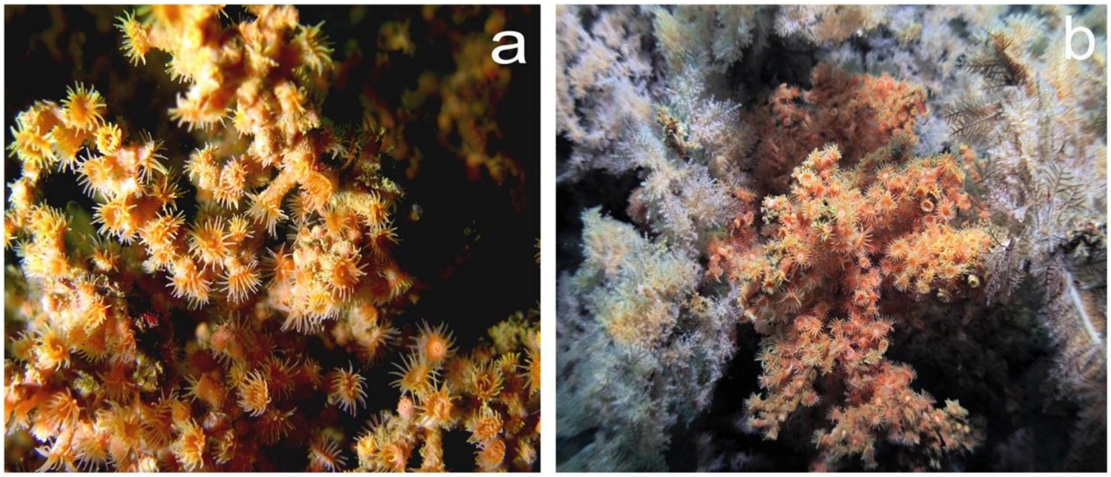

Figure. *In situ* pictures of *A. hickmani* at the MPA El Pelado, September 24, 2015. a) A closer view of the polyps of *A. hickmani;* b) The colony of *A. hickmani* on the branches of the black coral *M. panamensis.*

Morphological data: Alive/contracted polyps measured 6.0-12.0 / 2.0-4.0mm diameter and 2- 6 mm height (measured starting from the coenenchyme). Number of tentacles and mesenteries number approximately 36-40. Colours of the tentacles were yellow or orange, while the oral disk were yellow-cream and the column-coenenchyme were orange-cream. *A. hickmani* presented a

Cteniform endodermal type of sphincter muscle.[6] A relative amount of sand encrusted was visualized on the polyp's column and coenenchyme.

Molecular phylogeny. The results of molecular data were consistent with the literature data available. The mt 16S rDNA marker was successful sequenced for the sample 150924EP01-02 of *A. hickmani* (1061bp) this sequence was equal to the sequences from the original descriptions of *A. hickmani*. (EU333755, EU333756, both 582 bp) Only 1 bp was different from the *A. hickmani* EU333757 (582 bp). The Internal Transcribed Spacer region (ITS1, 5.8S and ITS2) was successful sequenced for *A. hickmani*, only one SNP and a polyA stretch of 9 successive bp instead of 7 bp in ITS1 remarkable the sequence of 150924EP01-02 *A. hickmani* (849 bp) from the original descriptions of *A. hickmani* (EU333797, EU333798, 703 bp).

[6] Swain, T. D., Schellinger, J. L., Strimaitis, A. M. & Reuter, K. E. Evolution of anthozoan polyp retraction mechanisms: convergent functional morphology and evolutionary allometry of the marginal musculature in order Zoanthidea (Cnidaria: Anthozoa: Hexacorallia). BMC Evol. Biol. 15, 123, (2015).

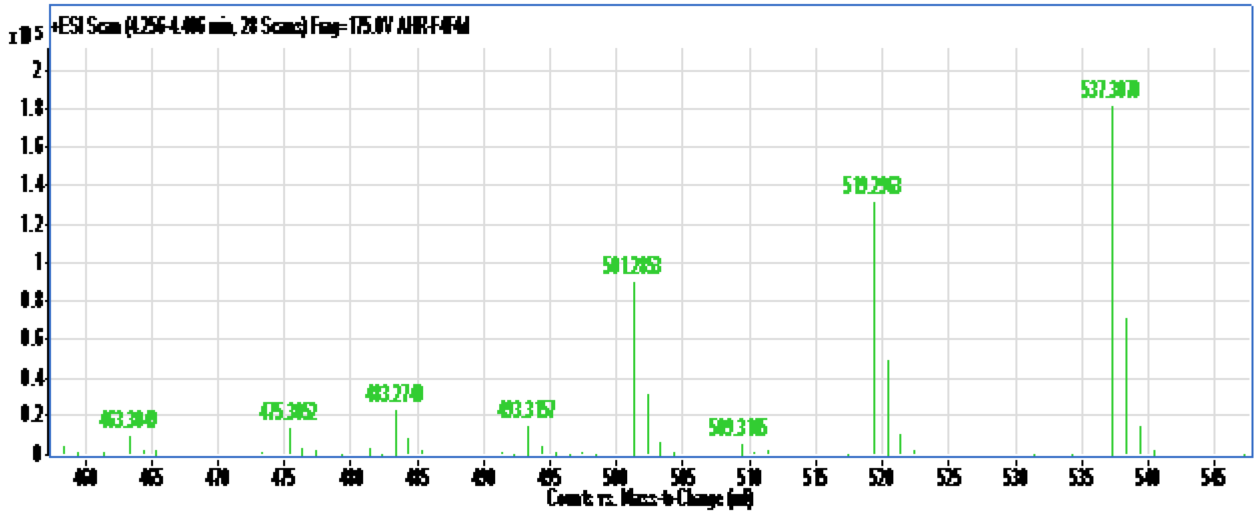

Figure S1. UHPLC-qToF analysis of **1** in (+)-ESI

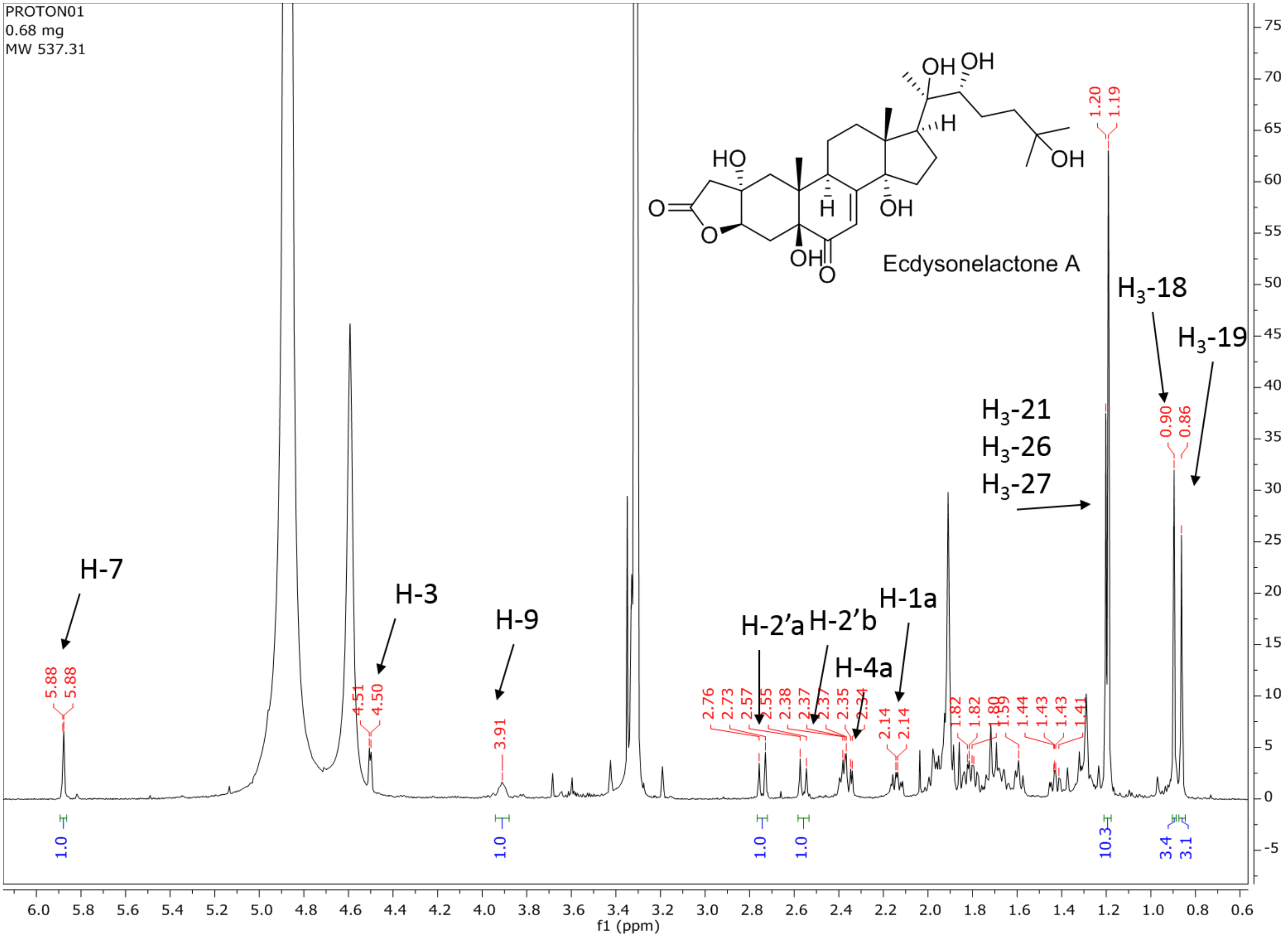

Figure S2. ^{1}H NMR spectrum of **1** at 600 MHz in CD_3OD

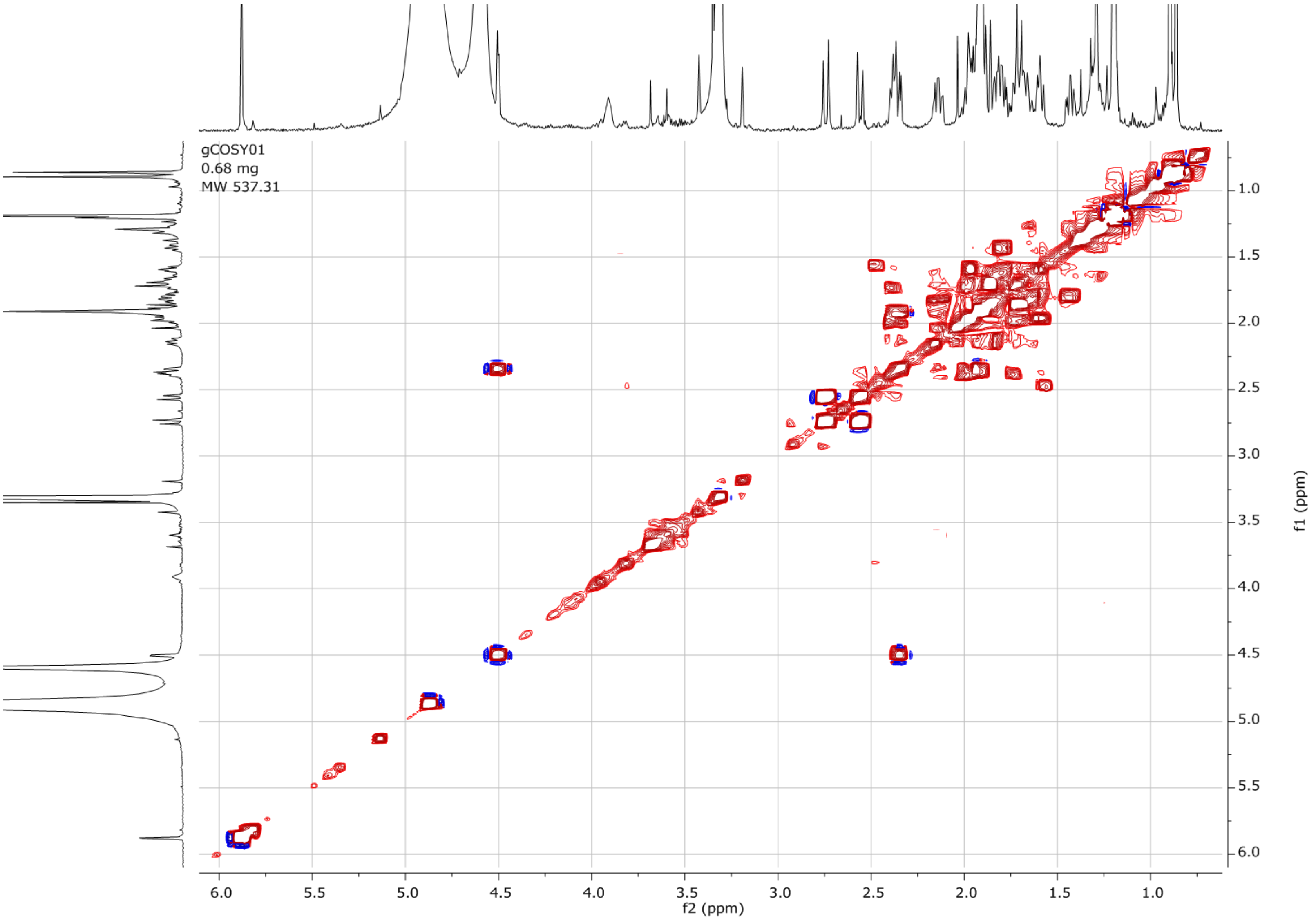

Figure S3. COSY NMR spectrum of **1** at 600 MHz in CD_3OD

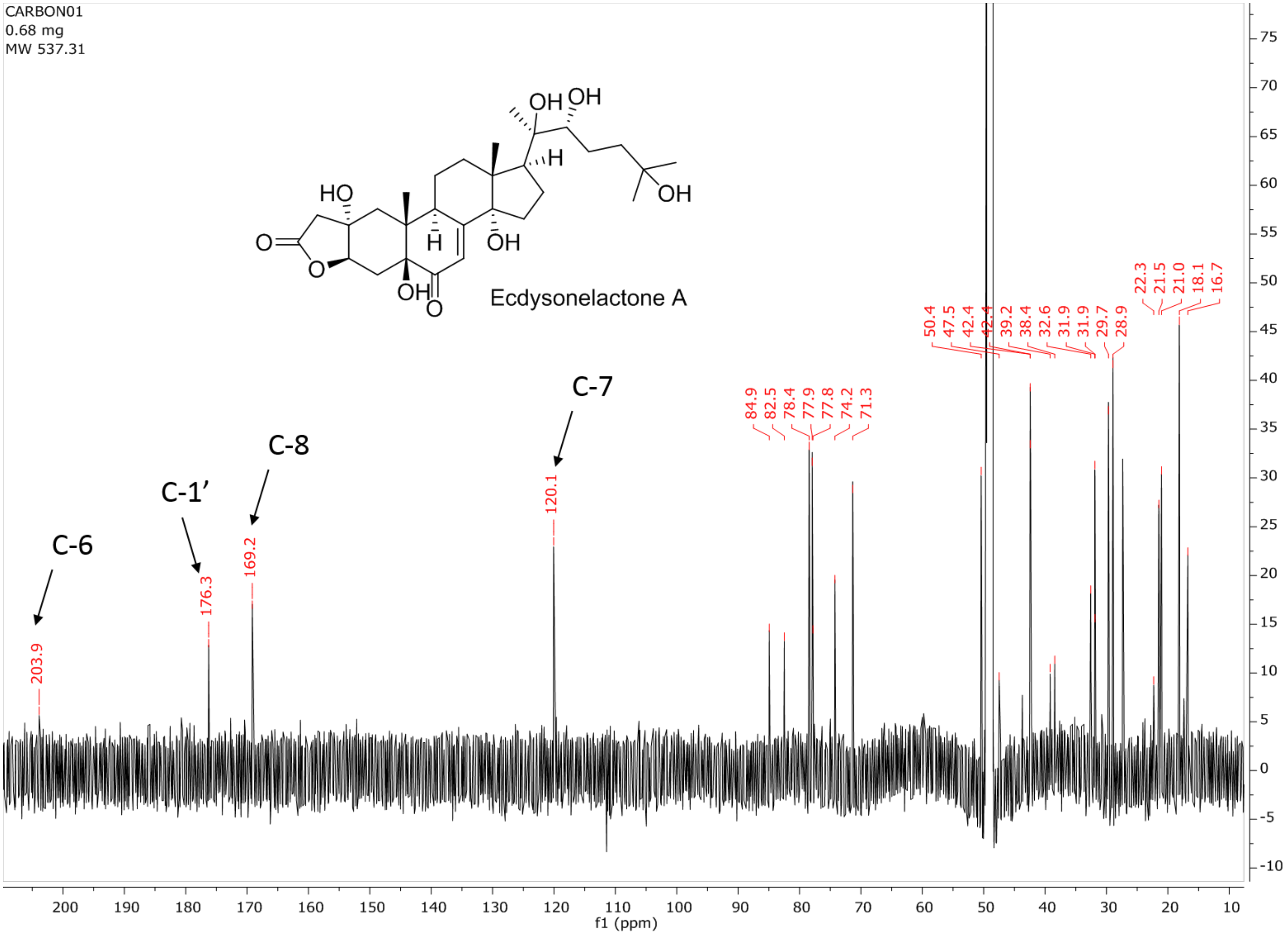

Figure S4. ^{13}C NMR spectrum of **1** at 150 MHz in CD_3OD

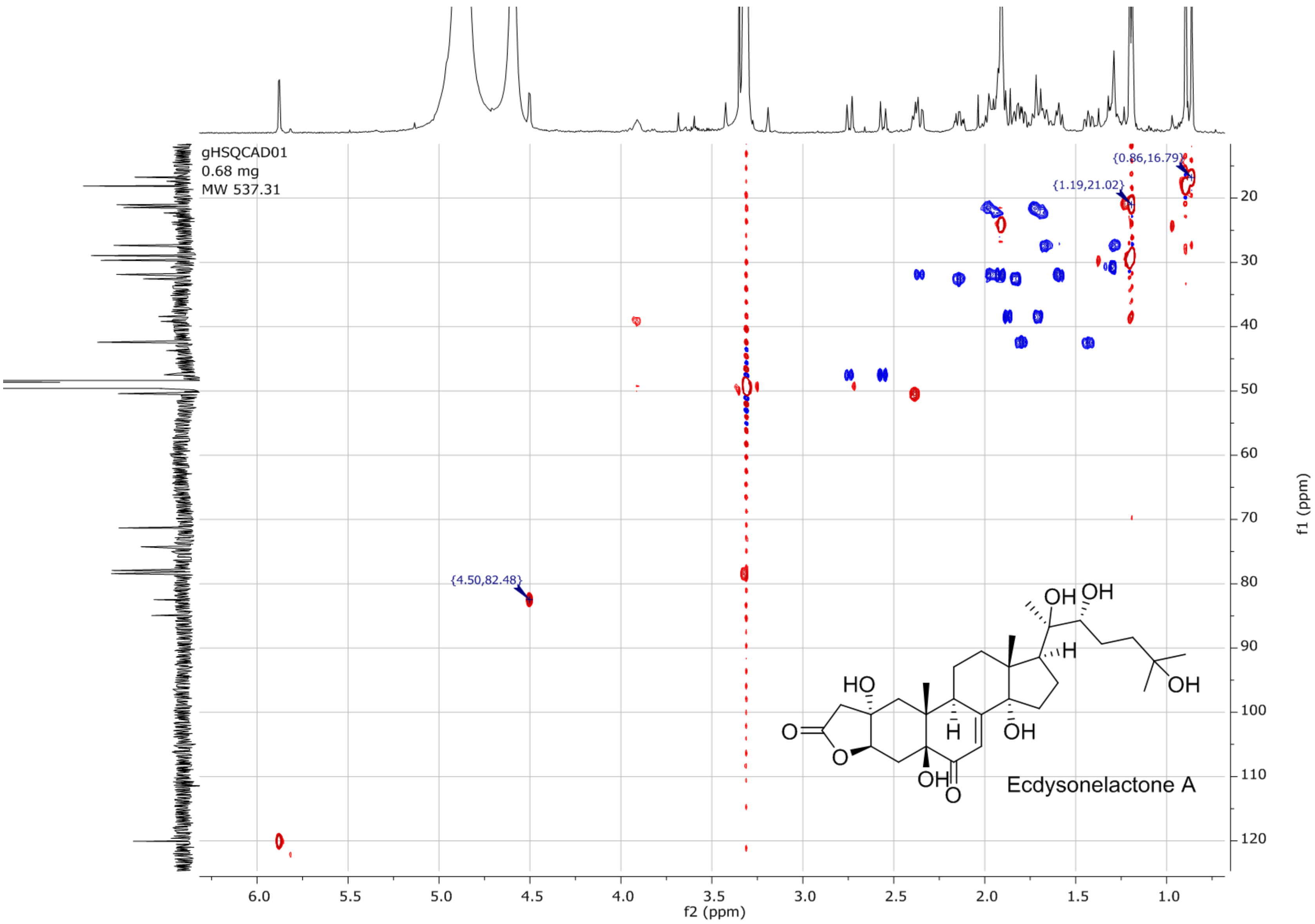

Figure S5. HSQC NMR spectrum of **1** at 600 MHz in CD_3OD

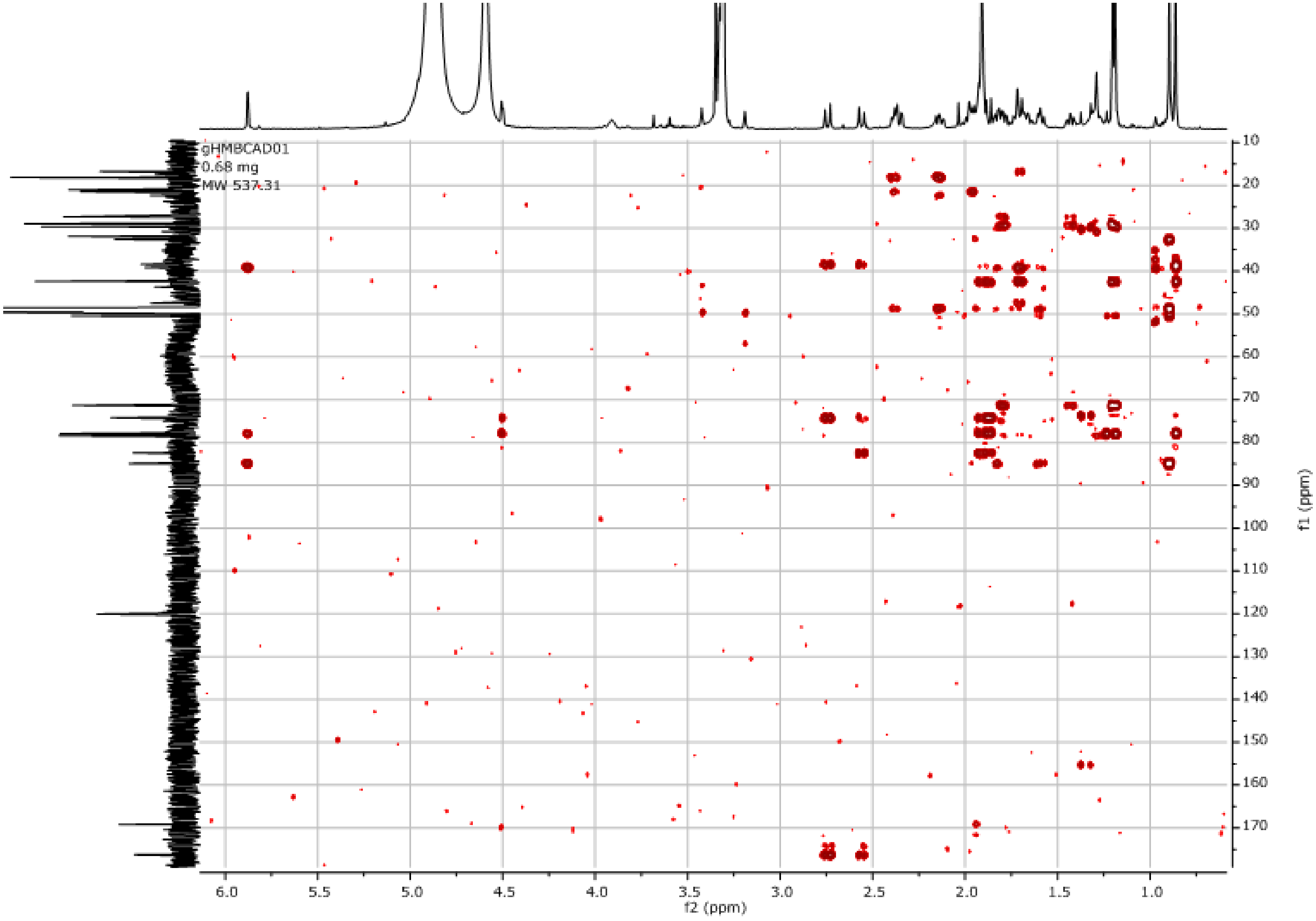

Figure S6. HMBC NMR spectrum of **1** at 600 MHz in CD_3OD

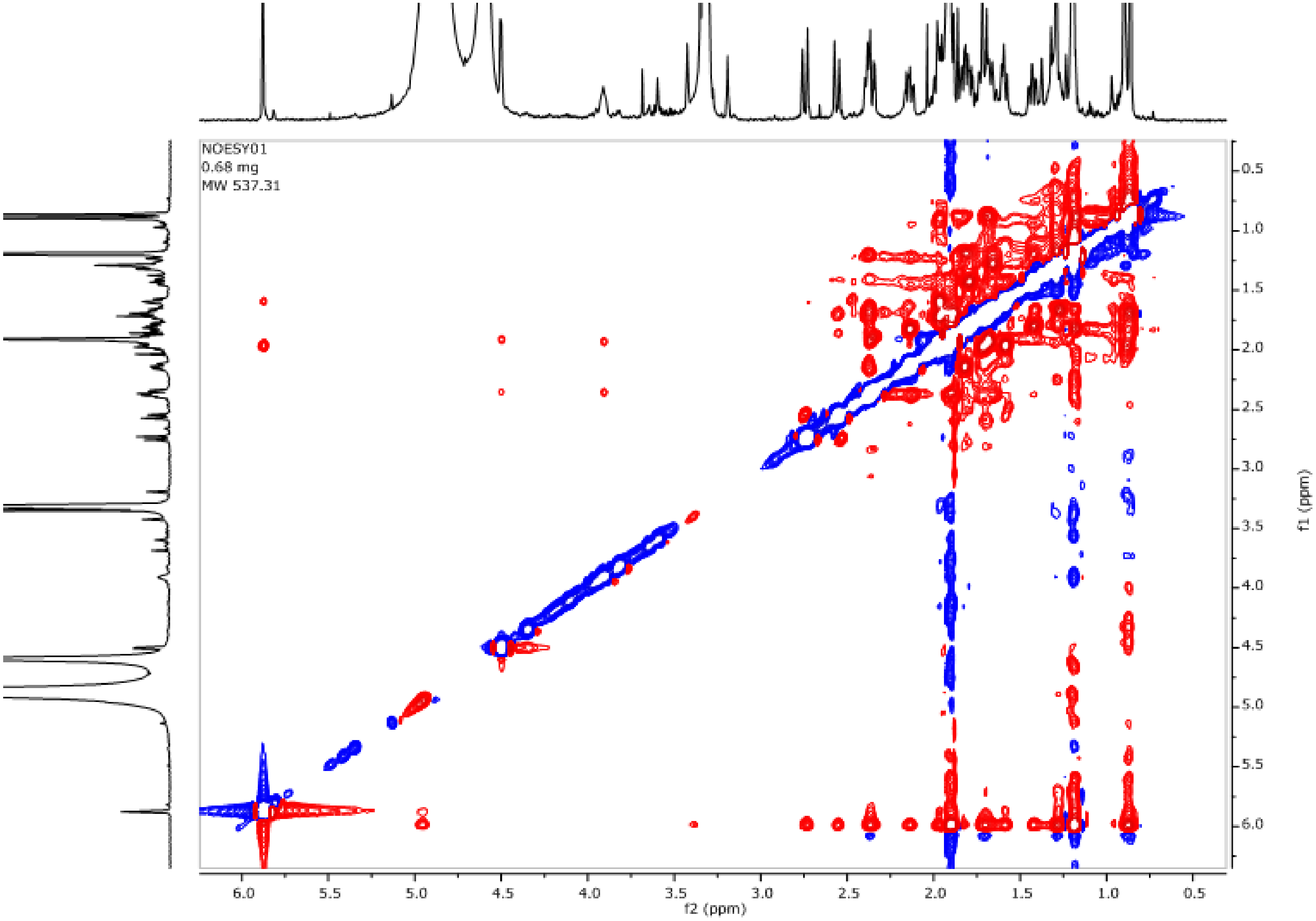

Figure S7. NOESY NMR spectrum of **1** at 600 MHz in CD_3OD

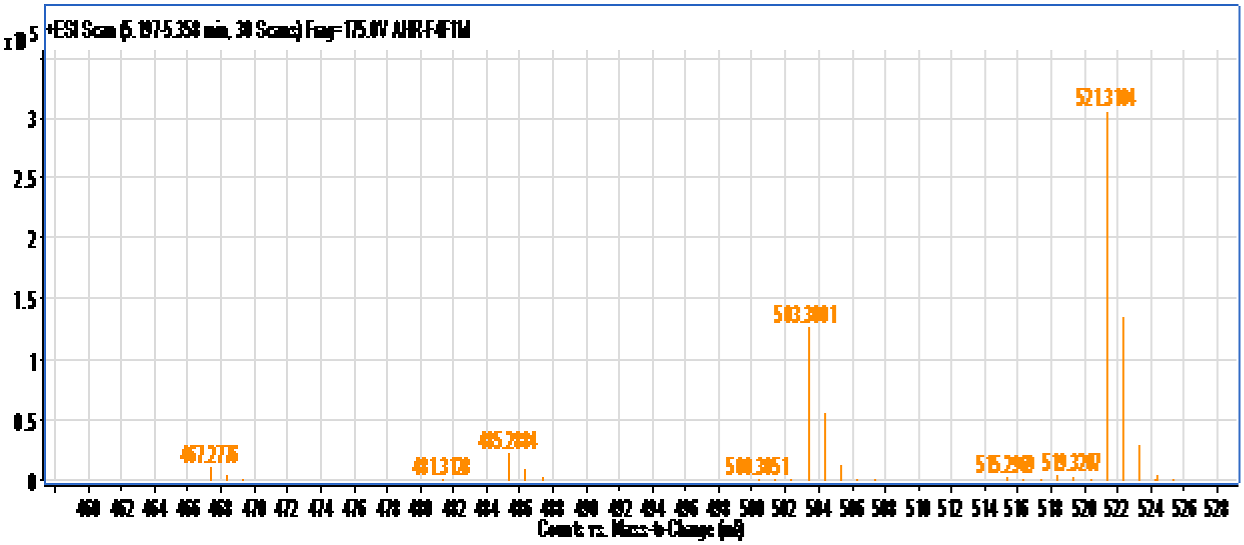

Figure S8. UHPLC-qToF analysis of **2** in (+)-ESI

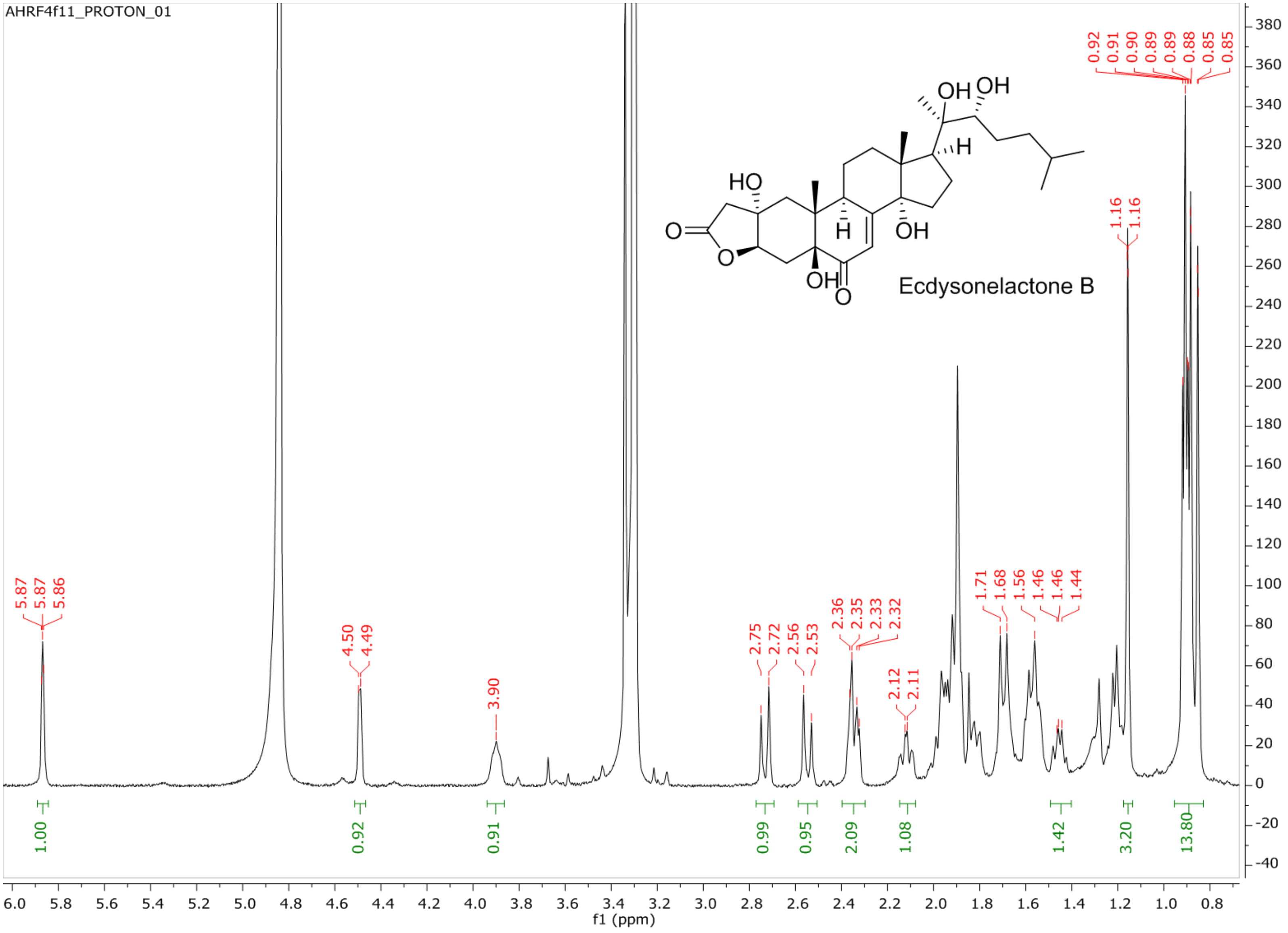

Figure S9. ^{1}H NMR spectrum of **2** at 600 MHz in CD_3OD

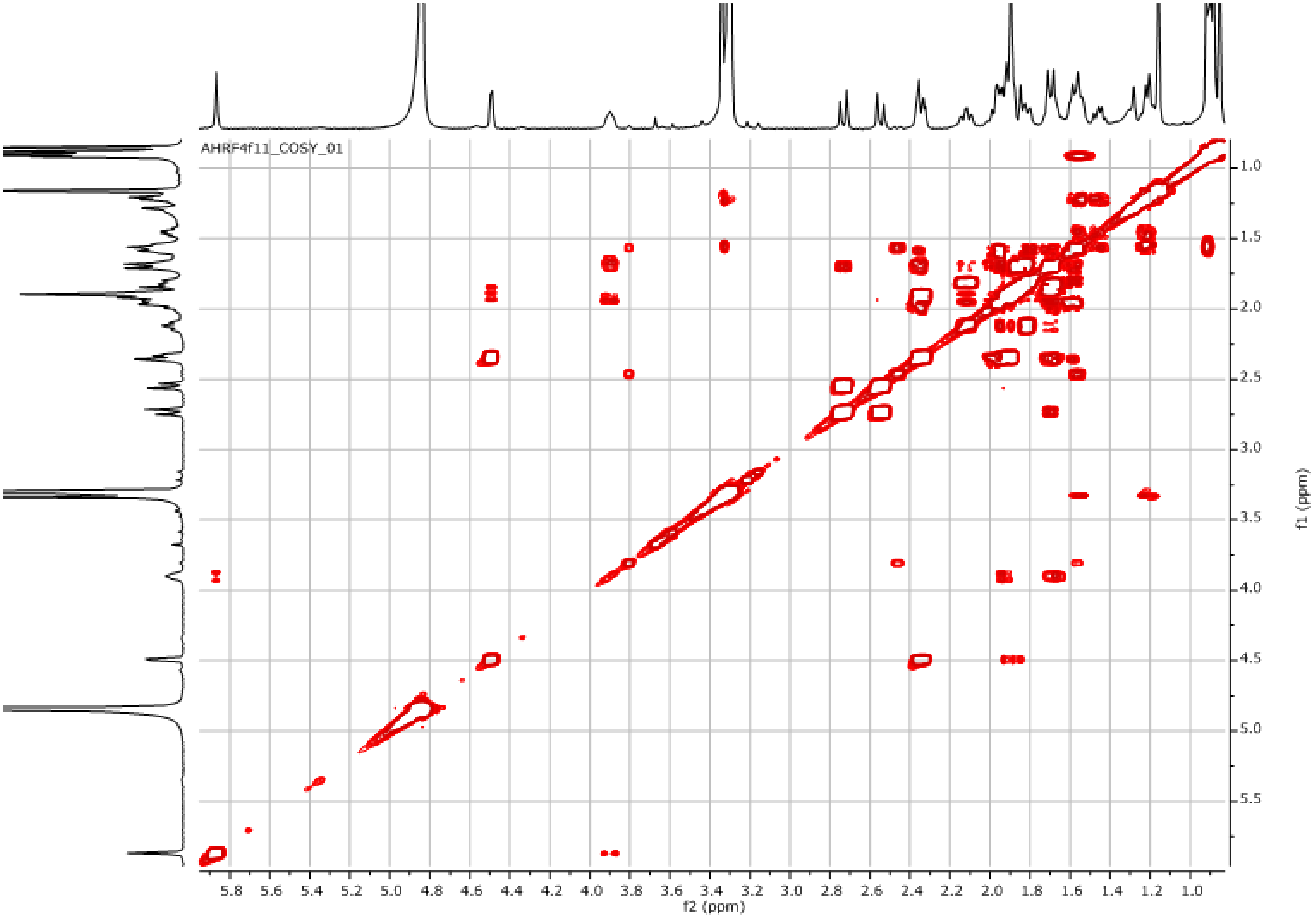

Figure S10. COSY NMR spectrum of **2** at 600 MHz in CD_3OD

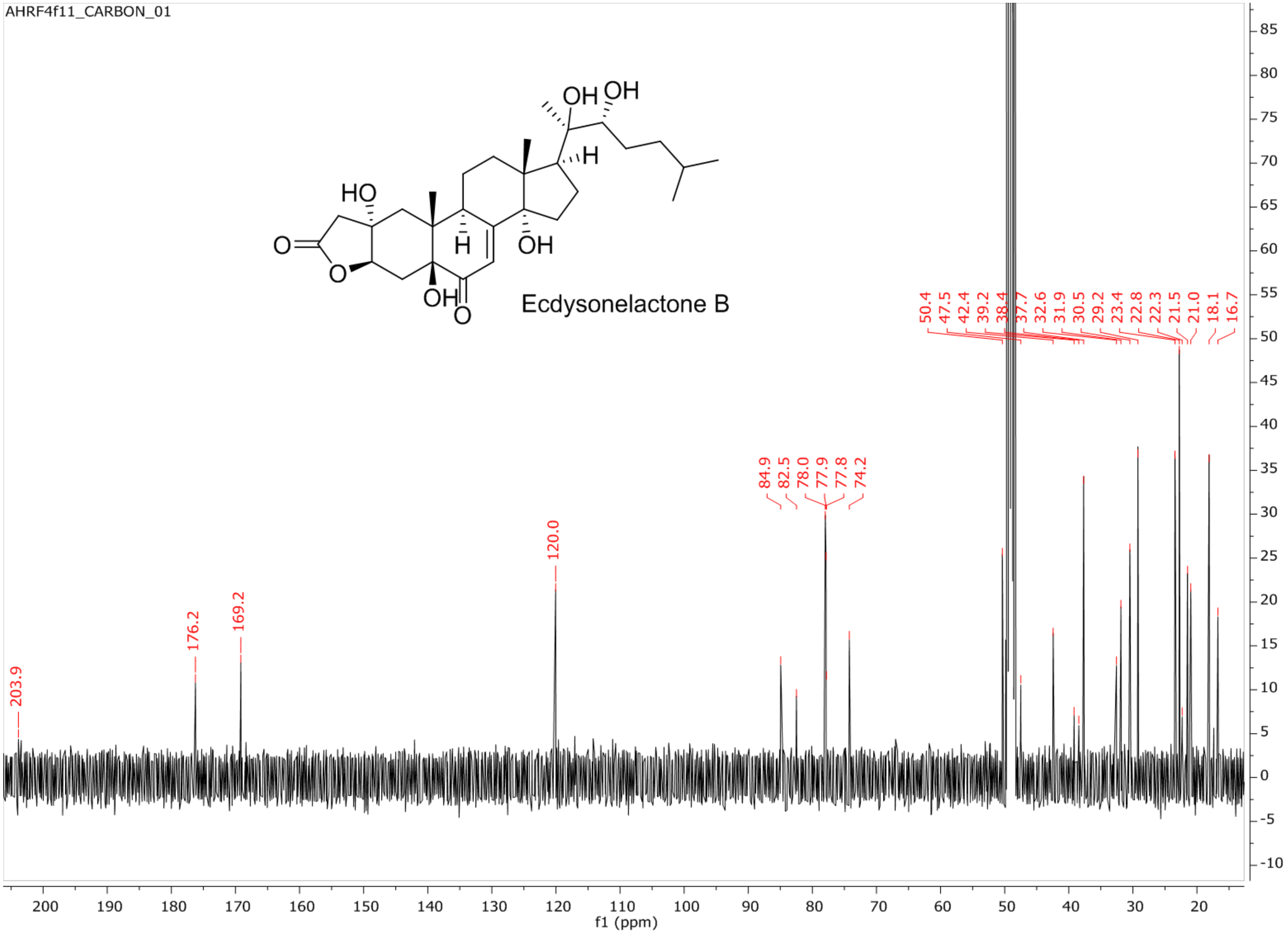

Figure S11. ${}^{13}C$ NMR spectrum of **2** at 150 MHz in CD_3OD

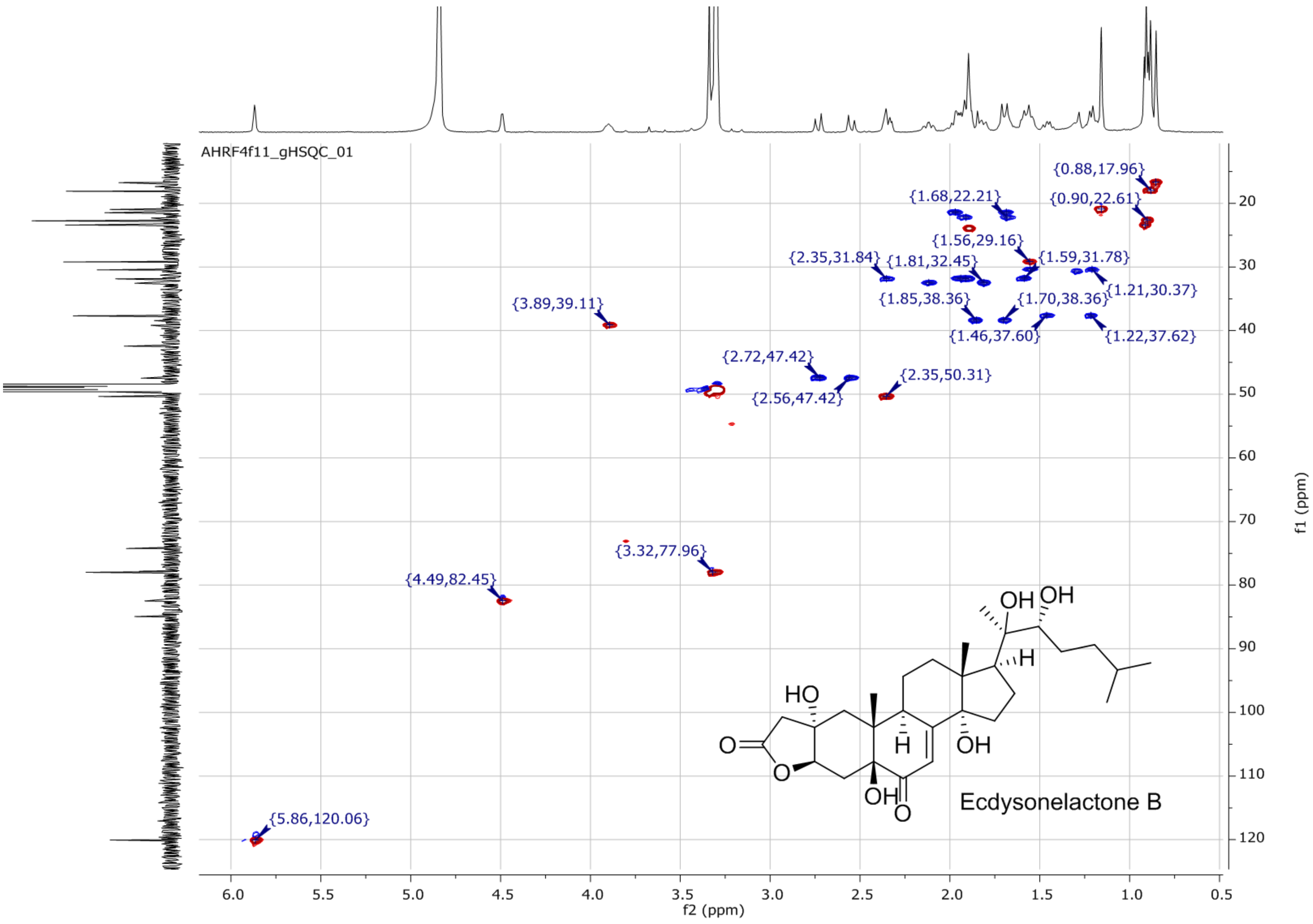
AHRF4f11_gHSQC_01
{0.88,17.96}
{1.68,22.21}
{0.90,22.61}
{1.56,29.16}
{2.35,31.84}
{1.81,32.45}
{1.59,31.78}
{1.21,30.37}
{3.89,39.11}
{1.85,38.36}
{1.70,38.36}
{1.46,37.60}
{1.22,37.62}
{2.72,47.42}
{2.35,50.31}
{2.56,47.42}
{3.32,77.96}
{4.49,82.45}
{5.86,120.06}
Ecdysonelactone B
f2 (ppm)
f1 (ppm)

Figure S12. HSQC NMR spectrum of **2** at 600 MHz in CD3OD

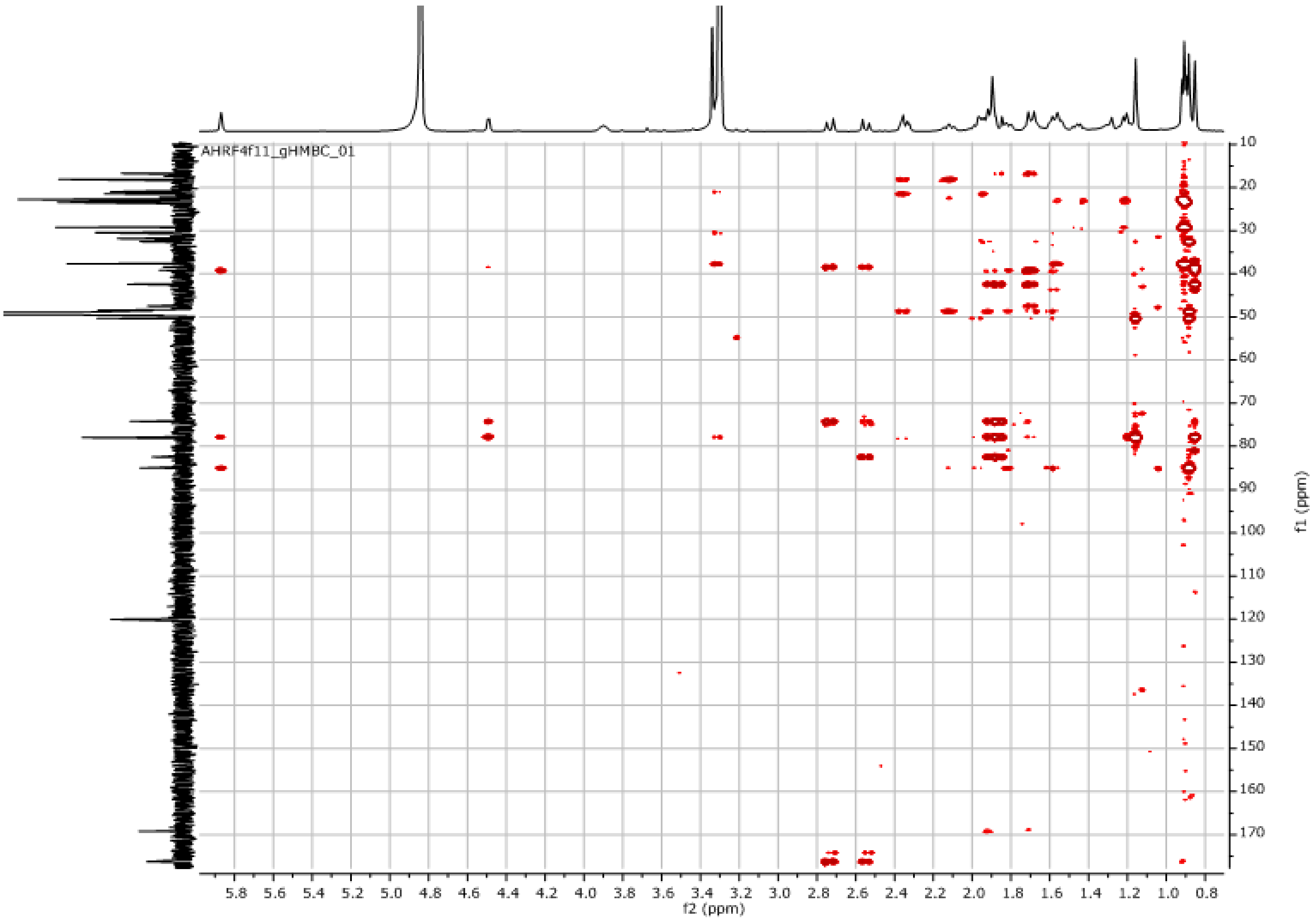

Figure S13. HMBC NMR spectrum of **2** at 600 MHz in CD3OD

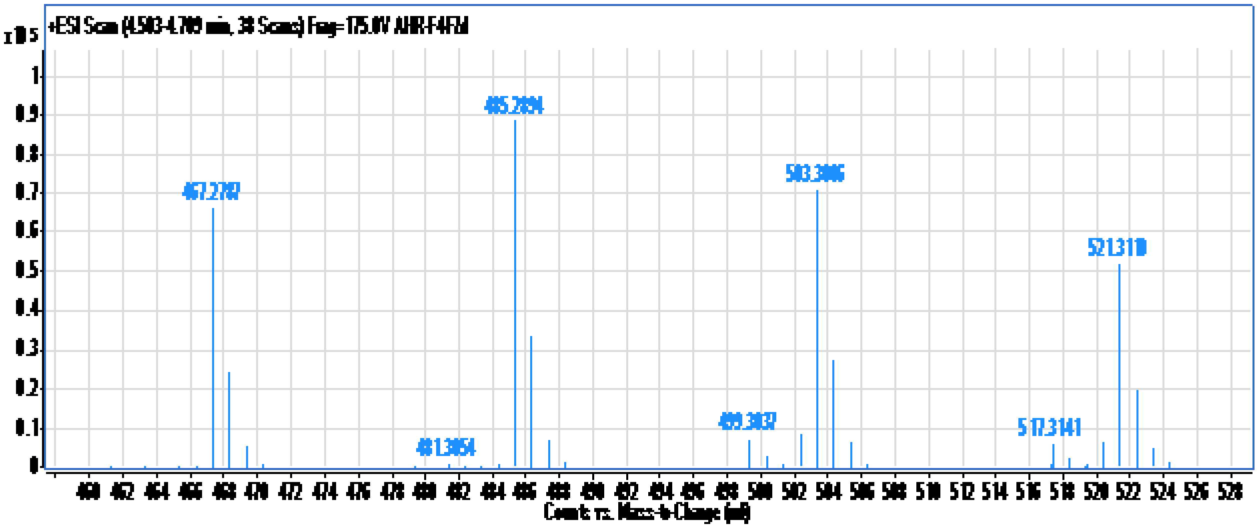

Figure S1. UHPLC-qToF analysis of **3** in (+)-ESI

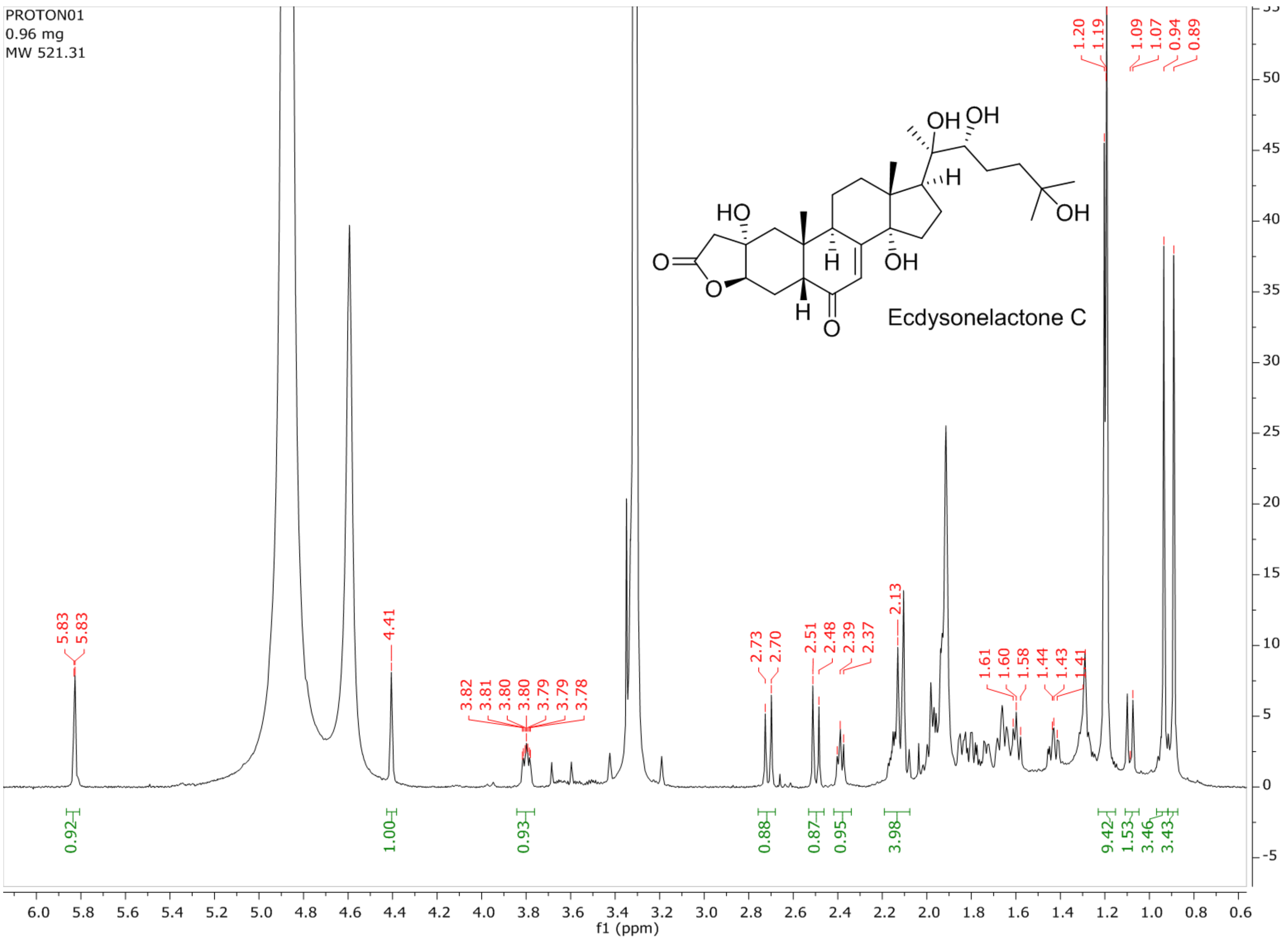

Figure S25. ^{1}H NMR spectrum of **3** at 600 MHz in CD_3OD

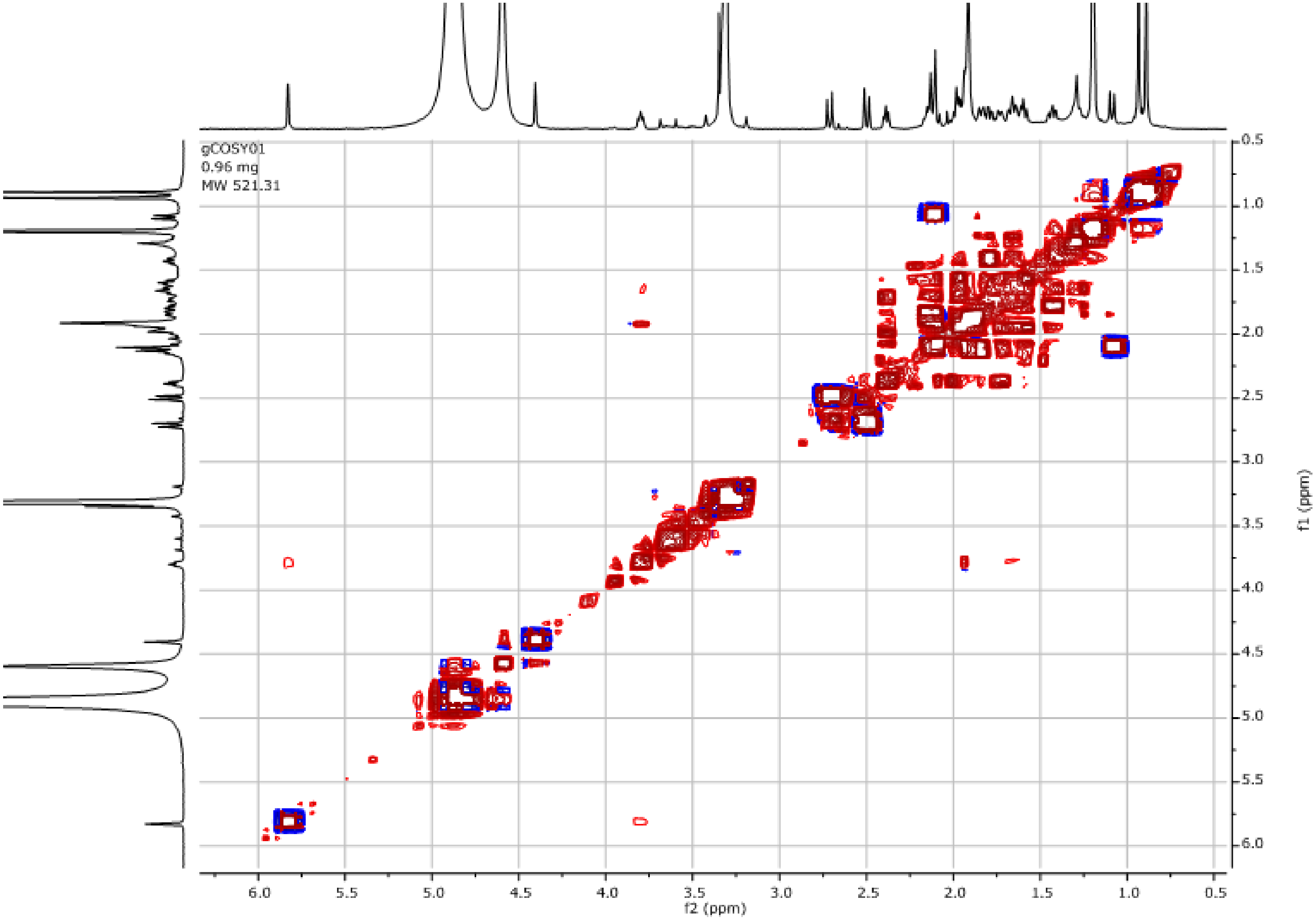

Figure S16. COSY NMR spectrum of **3** at 600 MHz in CD_3OD

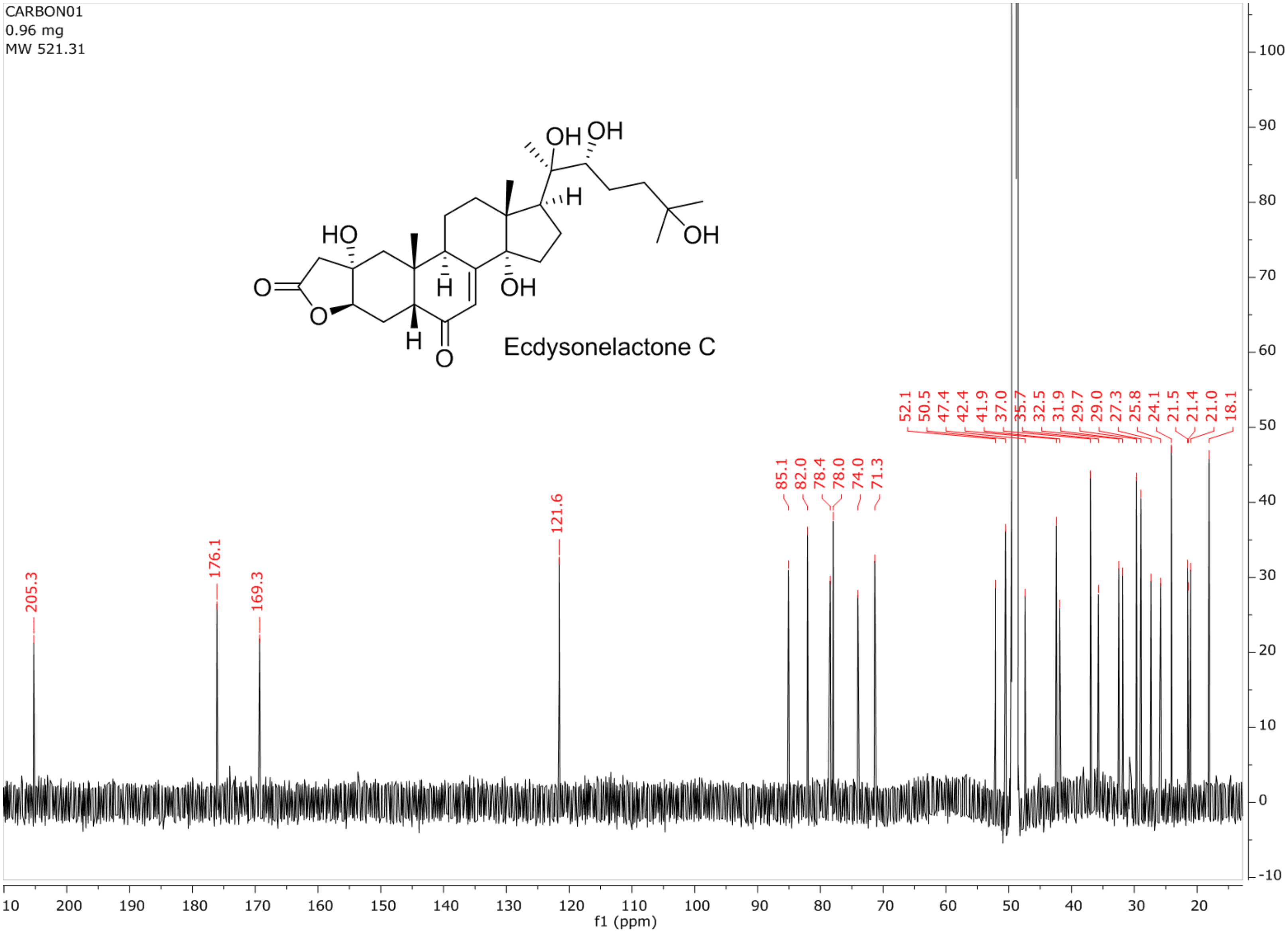

Figure S3. ^{13}C NMR spectrum of **3** at 150 MHz in CD_3OD

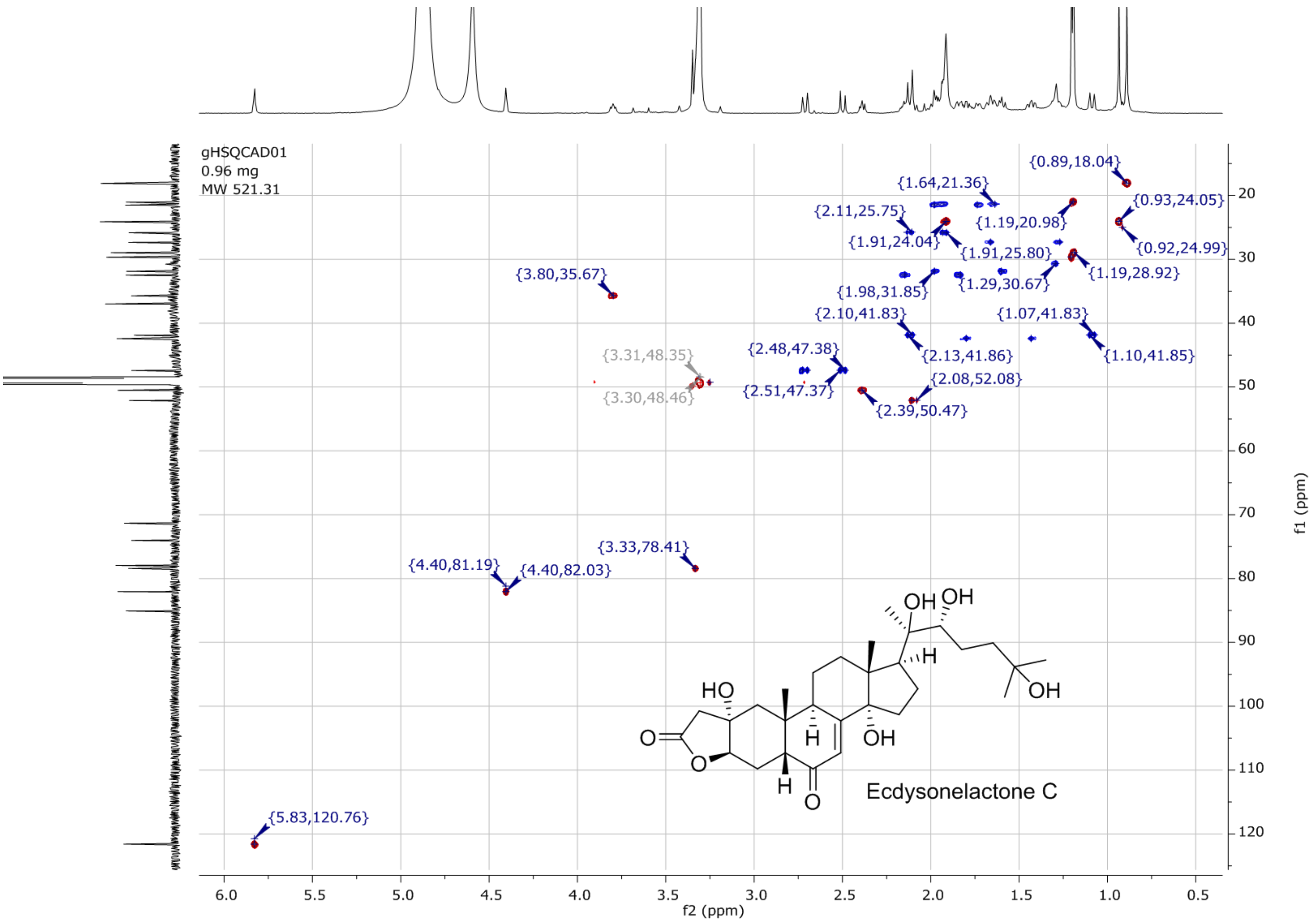

Figure S18. HSQC NMR spectrum of **3** at 600 MHz in CD_3OD

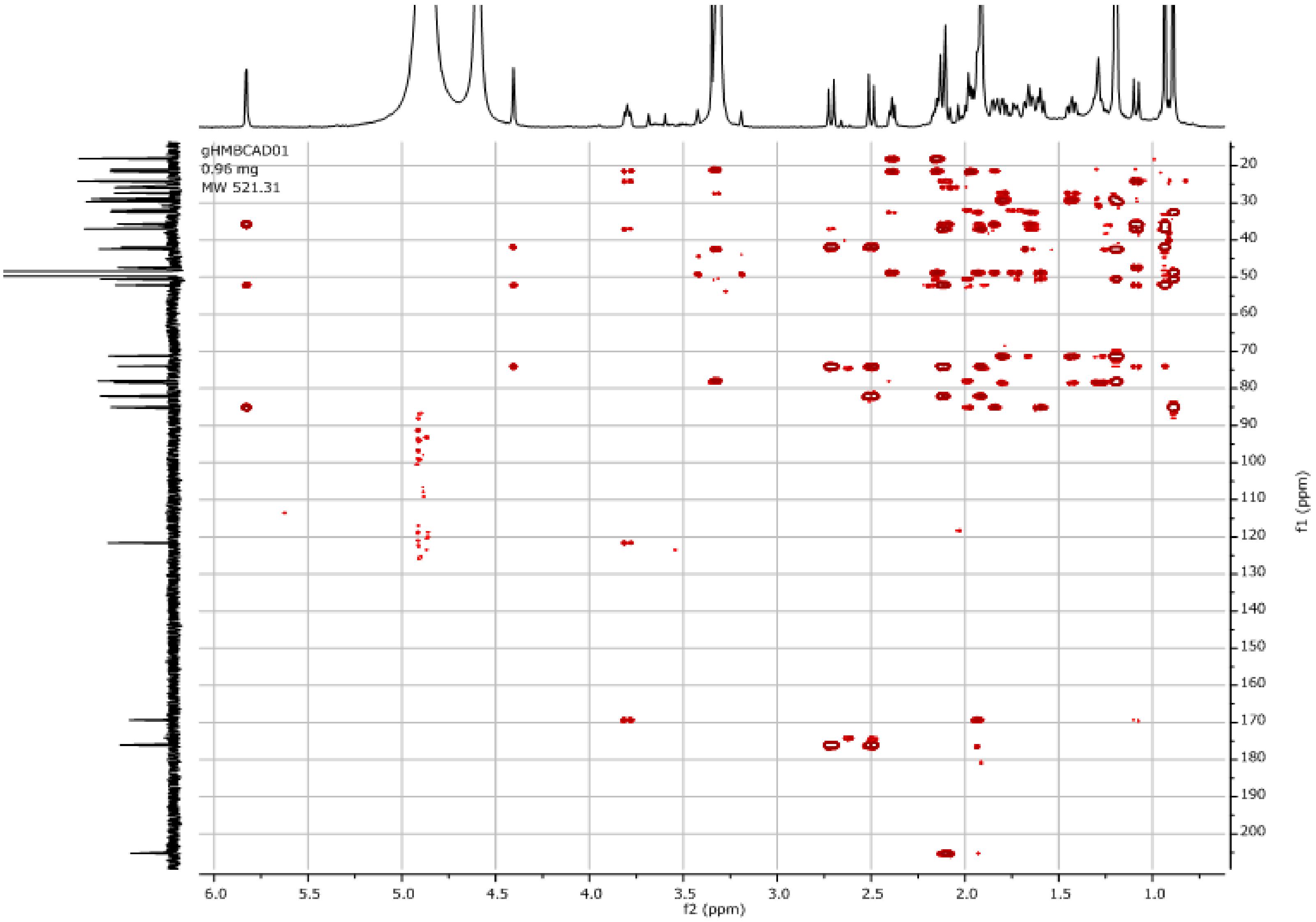

Figure S4. HMBC NMR spectrum of **3** at 600 MHz in CD_3OD

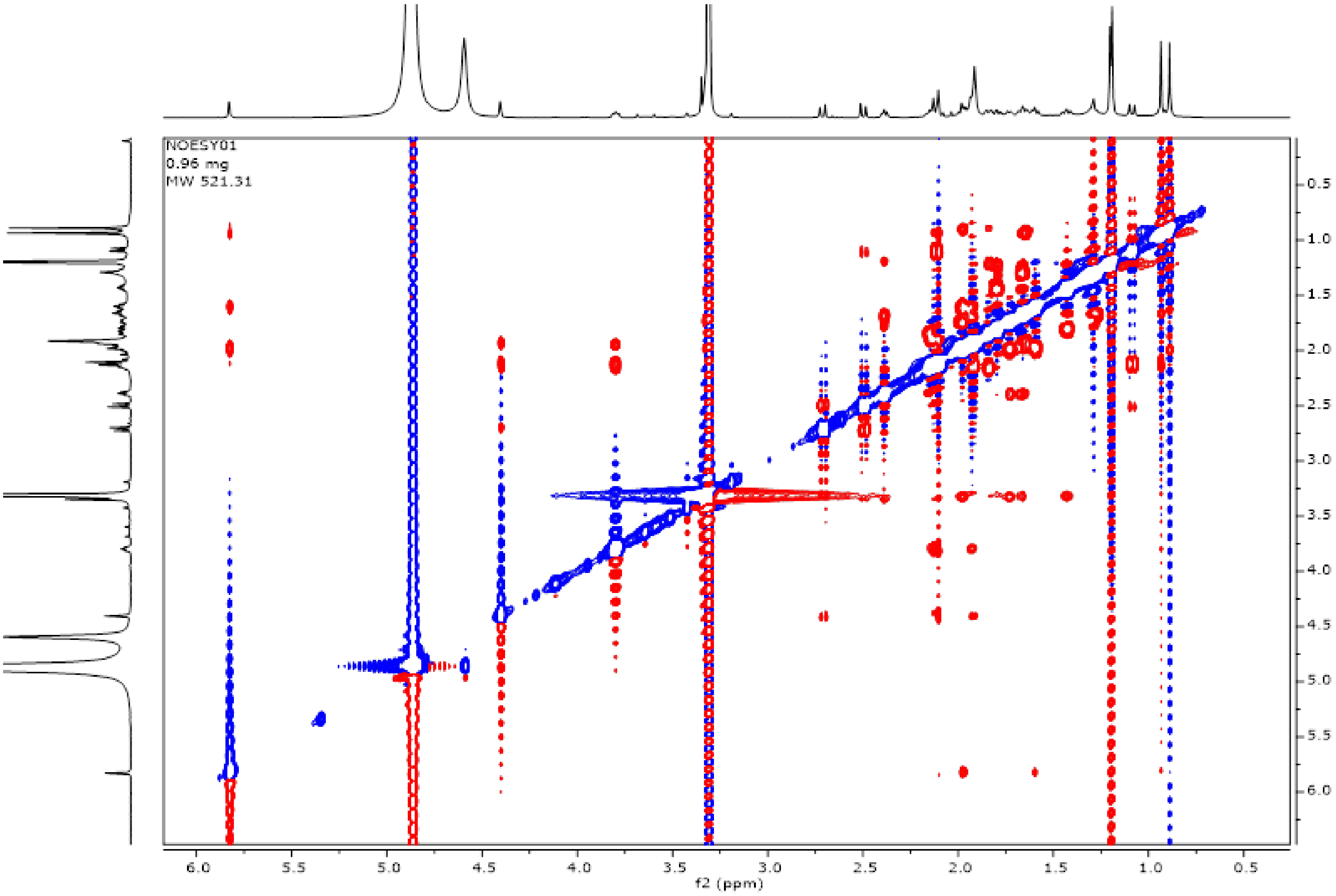

Figure S20. NOESY NMR spectrum of **3** at 600 MHz in CD_3OD

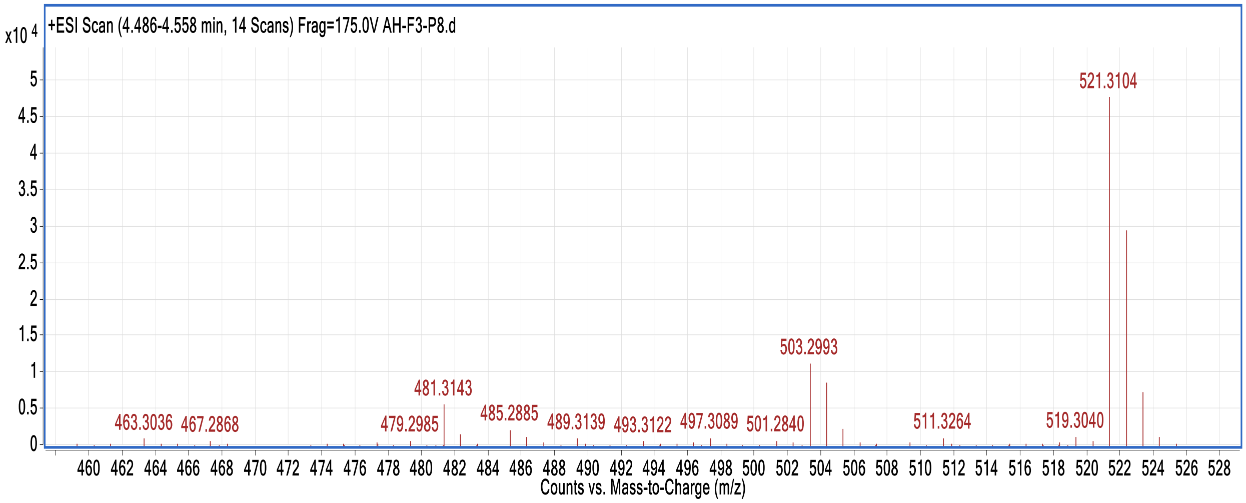

Figure S21. (+)-HRESIMS analysis of **4**.

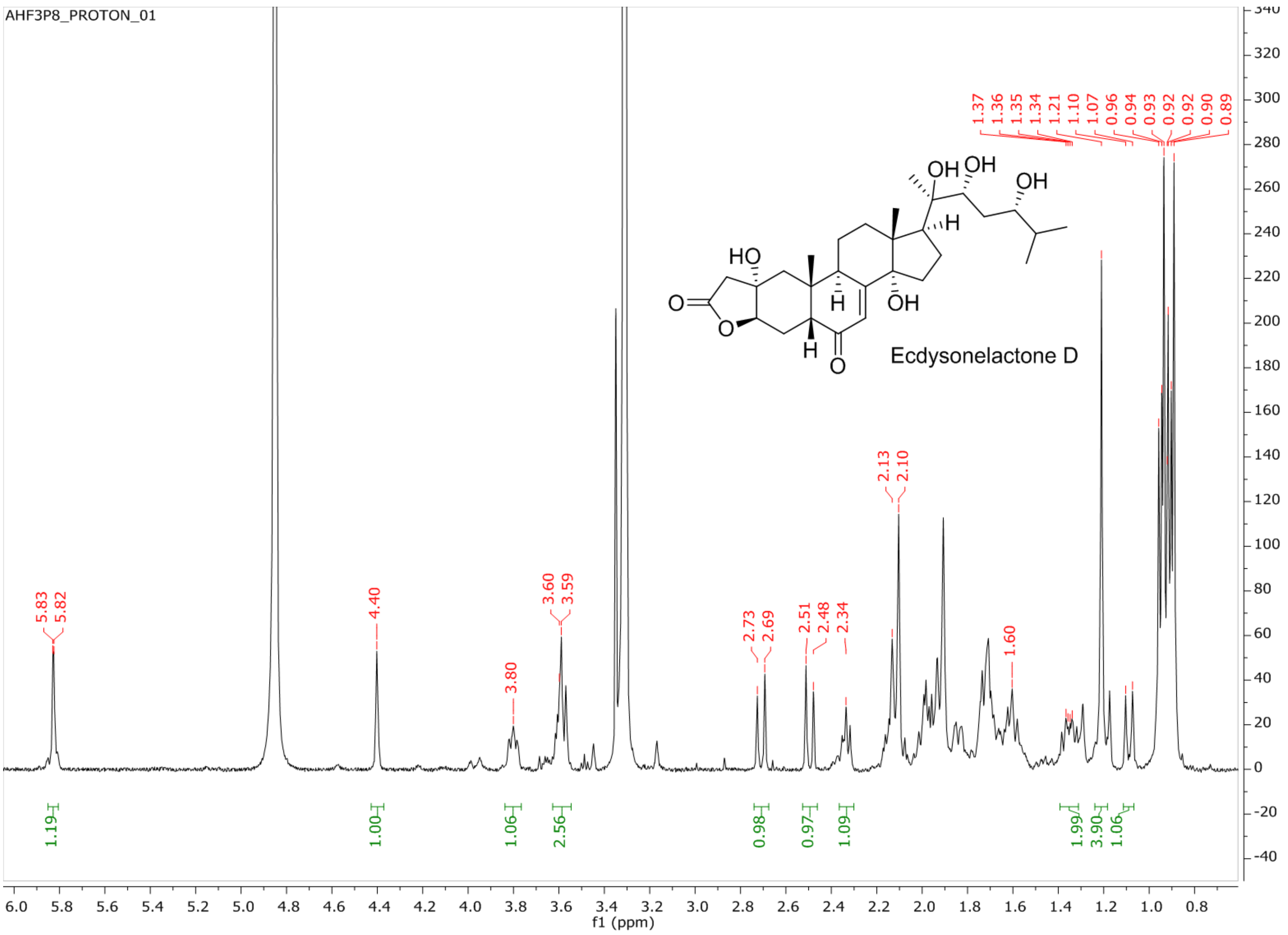

Figure S52 ^{1}H NMR spectrum of **4** at 600 MHz in CD_3OD

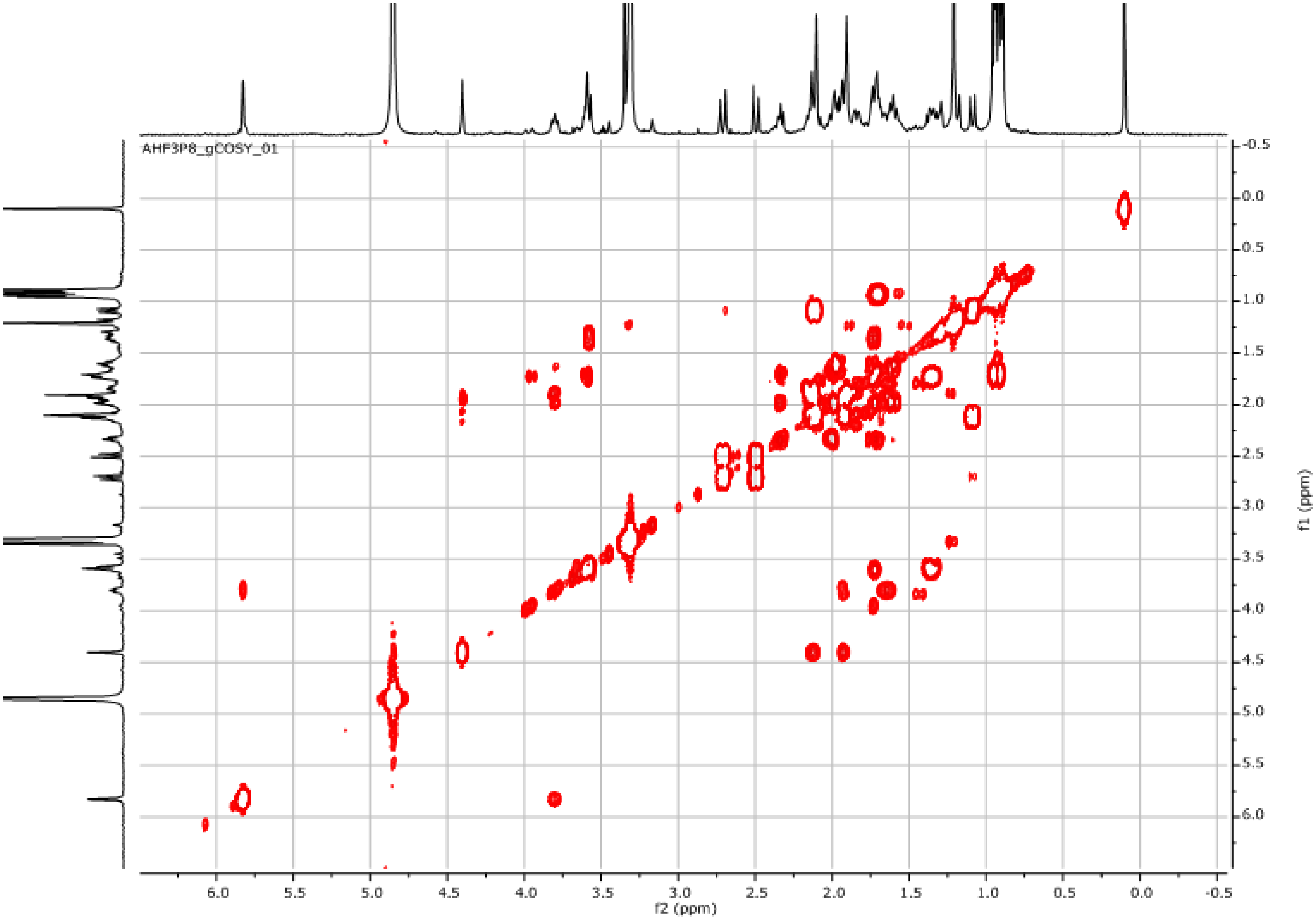

Figure S26. COSY NMR spectrum of **4** at 600 MHz in CD_3OD

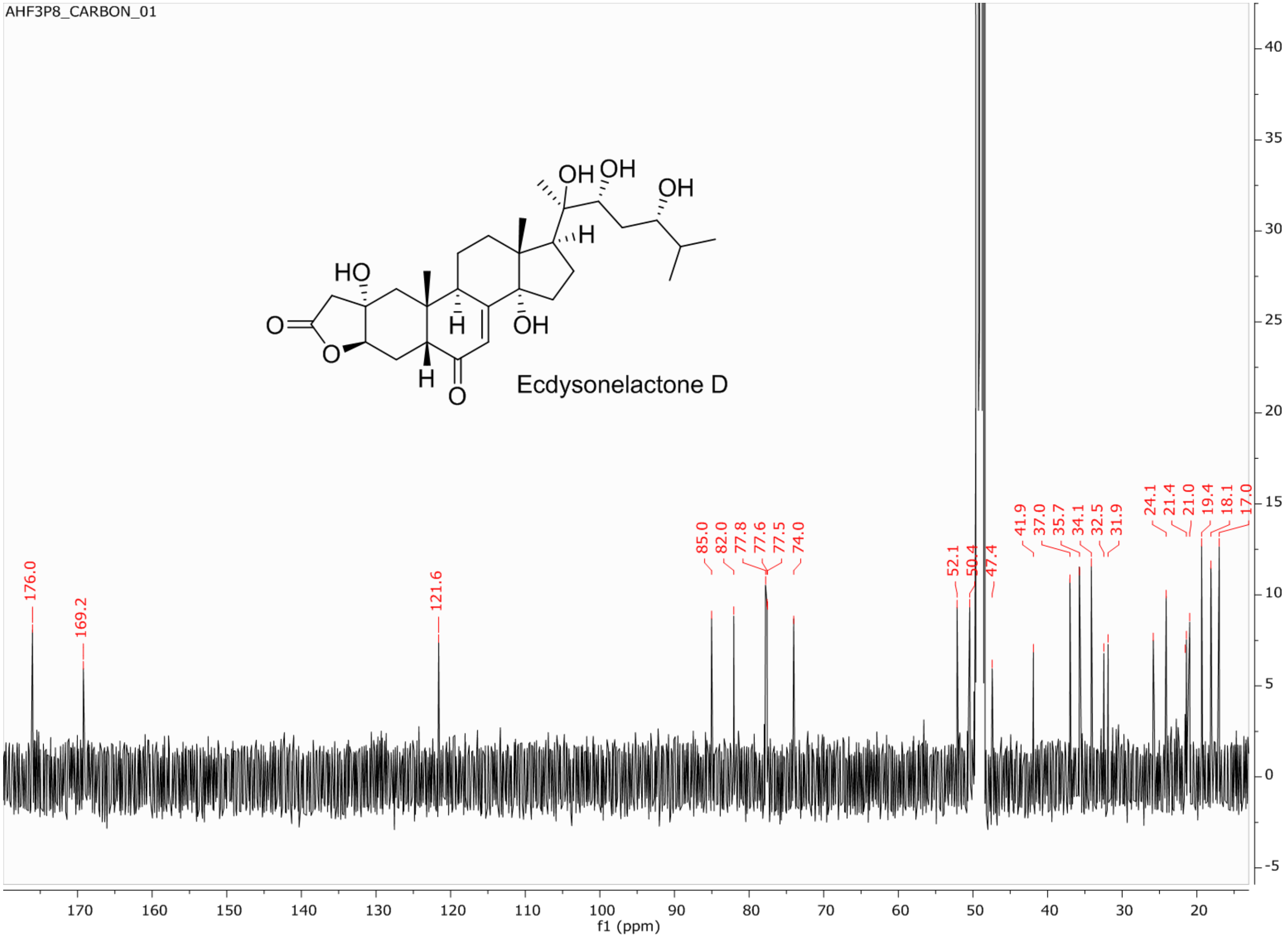

Figure S27. ^{13}C NMR spectrum of **4** at 150 MHz in CD_3OD

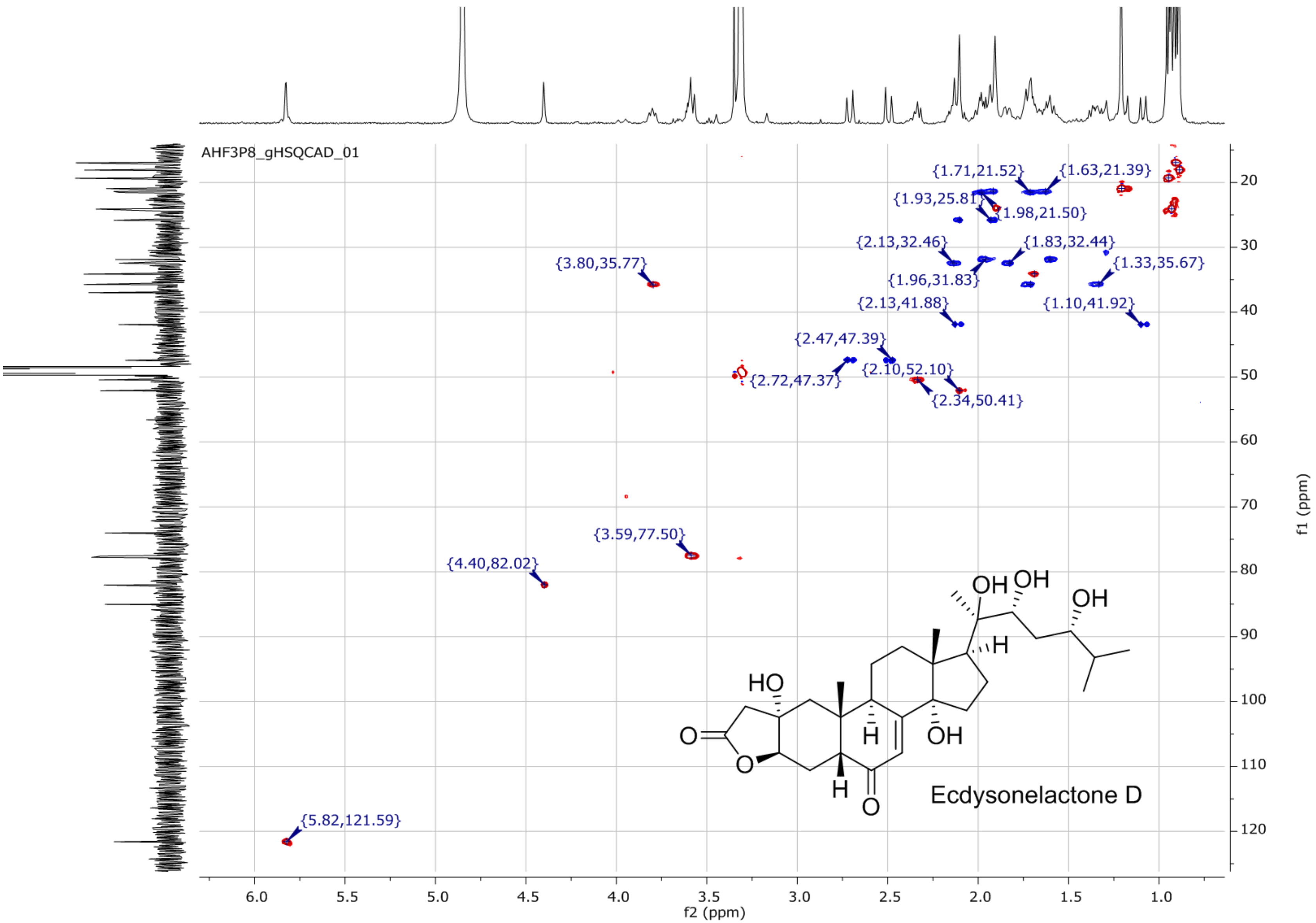

Figure S25. HSQC NMR spectrum of **4** at 600 MHz in CD_3OD

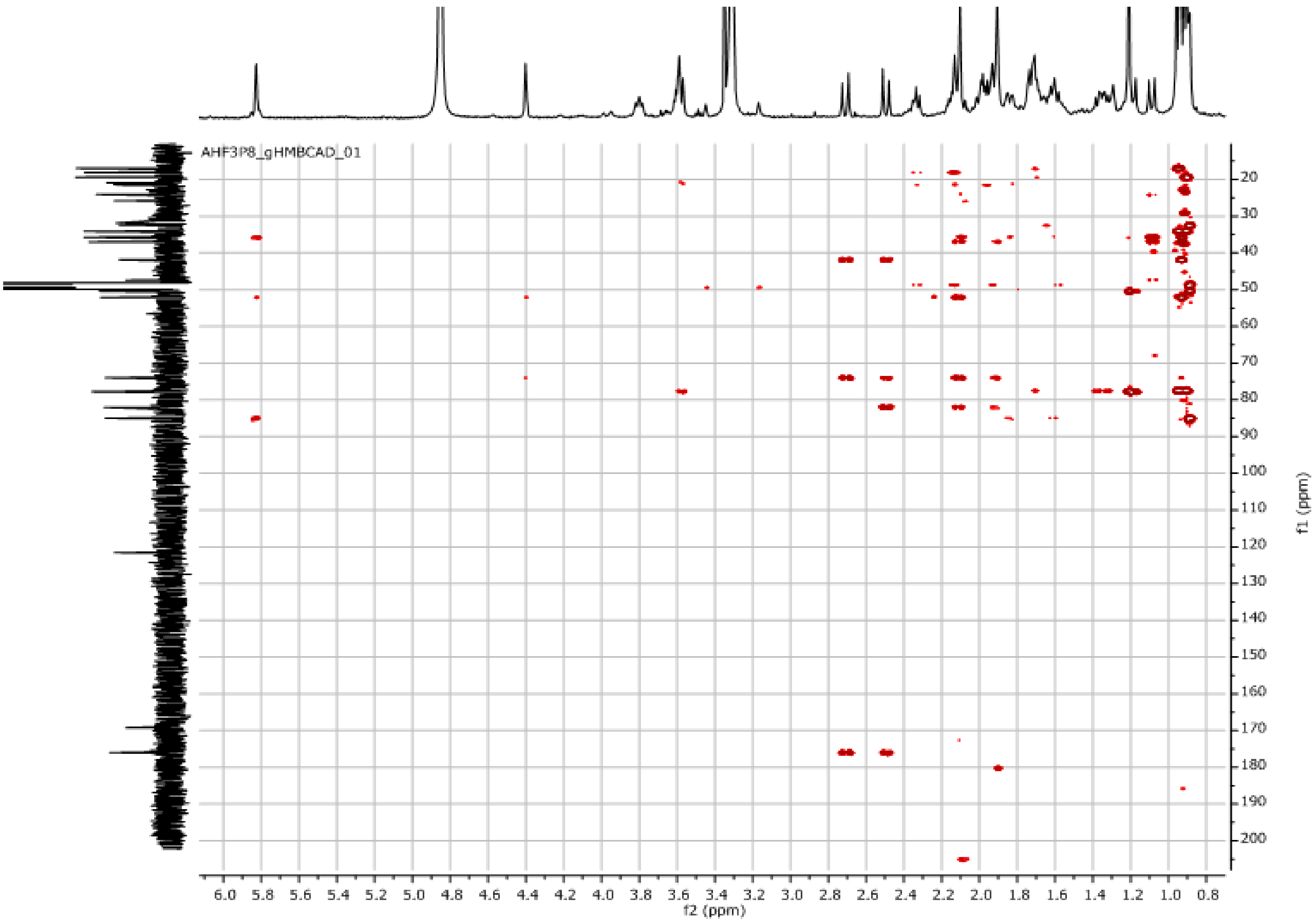

Figure S26. HMBC NMR spectrum of **4** at 600 MHz in CD_3OD

Computational details.

The most stable conformer was generated using the MMFF94 force field (MarvinView (version 6.2.2), calculation module developed by ChemAxon) and the geometry described in Ohta *et al.*[7] as initial input. The ^{3}JH,H couplings were predicted using MSpin[8] for each diasteroisomers.

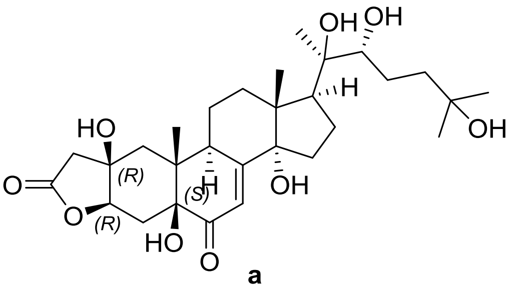

a

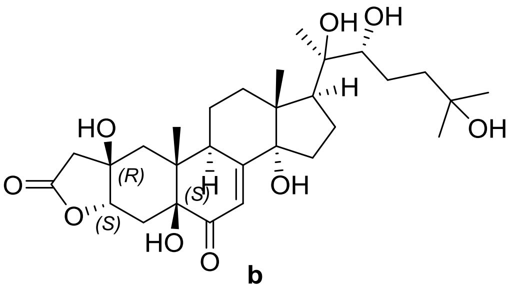

b

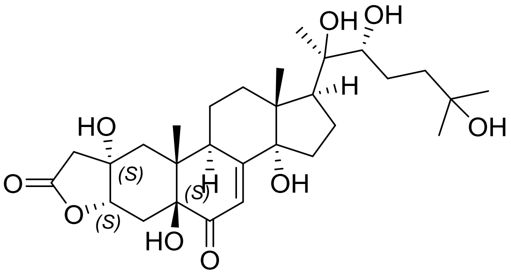

c

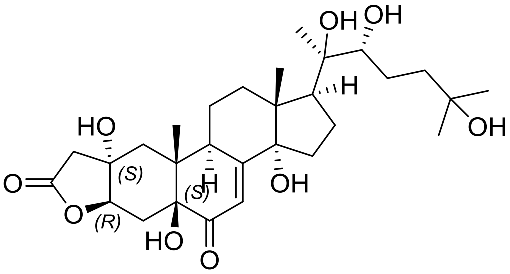

d

Cartesian coordinates of the most stable conformers of each diastereoisomer of ***1****.*

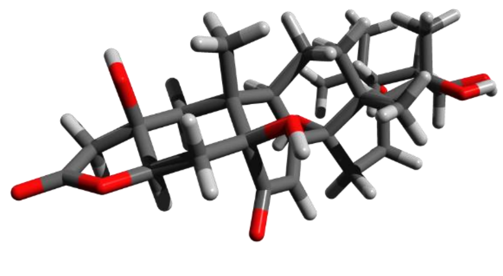				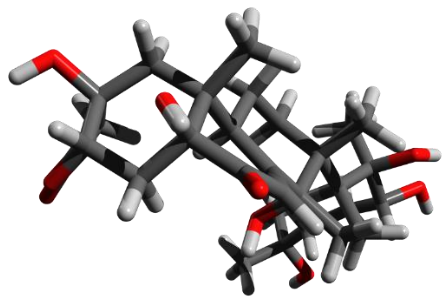			
C	-3.41240	2.21550	10.18410	C	-1.54260	4.37680	10.00810
C	-2.21870	2.55190	11.05650	C	-2.35740	3.17150	9.55550
C	-1.15700	3.32170	10.28750	C	-1.58570	2.12250	8.75870
C	-0.64380	2.45930	9.09710	C	-0.29340	1.72110	9.47640
C	-1.80120	1.97080	8.15020	C	0.66340	2.93200	9.66170

7 Shinji Ohta, Jian-Ru Guo, Yoshikazu Hiraga, Takayuki Suga 24-Epi-pterosterone: A novel phytoecdysone from the roots of Athyrium yokoscense Phytochemistry 1996, 41(3), 745-747. DOI: 10.1016/0031-9422(95)00688-5
8 A. Navarro-Vázquez MSpin-RDC. A program for the use of Residual Dipolar Couplings for Structure Elucidation of Small Molecules. Magn. Reson. Chem. 2012, 50, S73-S79. DOI: 10.1002/mrc.3905

C	-2.96200	1.32250	9.02490	C	-0.09770	4.06100	10.43160
C	-4.34470	1.67220	11.24170	C	-1.70480	5.37730	8.87780
C	-4.01300	2.61690	12.38280	C	-2.98920	4.93990	8.24100
O	-2.79050	3.21590	12.17430	O	-3.38320	3.72330	8.71630
C	0.15910	1.26700	9.65720	C	0.40350	0.60020	8.69690
C	0.43640	0.11610	8.78300	C	1.25570	0.96580	7.54740
C	-0.18450	-0.04420	7.60790	C	1.58000	2.24790	7.31090
C	-1.30550	0.87060	7.10410	C	1.19770	3.40600	8.24940
C	0.21830	-1.12400	6.62570	C	2.25790	2.68510	6.03270
C	0.68260	-0.50930	5.26370	C	3.49570	3.59550	6.28620
C	-0.49260	0.30970	4.69670	C	3.00090	4.85110	7.03520
C	-0.96000	1.39820	5.68400	C	2.31070	4.48780	8.35930
C	1.34910	-2.11020	6.93600	C	2.79150	1.66450	5.02520
C	1.75150	-2.67580	5.56260	C	3.72790	2.48290	4.12050
C	1.03910	-1.81860	4.47470	C	3.96220	3.86080	4.81140
O	-4.70800	2.79690	13.36940	O	-3.60400	5.56970	7.39590
O	-4.03920	3.38880	9.65900	O	-2.16800	4.97850	11.16290
C	-2.37180	3.19370	7.37180	C	1.85360	2.50710	10.57610
C	1.91290	0.42350	5.42600	C	4.58180	2.87350	7.12400
O	-0.96500	-1.92300	6.43720	O	1.23860	3.43580	5.33980
C	1.82410	-1.77690	3.12760	C	5.36990	4.45420	4.50320
C	2.20740	-3.19790	2.57120	C	5.63570	4.64550	2.96460
C	1.12750	-4.04100	1.87140	C	5.07520	5.89780	2.26810
C	-0.02780	-4.49700	2.76460	C	3.54950	6.01060	2.23240
C	-0.88220	-5.64120	2.18420	C	3.01350	7.02980	1.20500
C	-1.96080	-6.03510	3.19930	C	1.48160	7.03310	1.23160
C	-1.52730	-5.26160	0.85050	C	3.54640	8.44210	1.45490
O	3.26430	-2.98290	1.59850	O	7.07800	4.69270	2.80390
C	1.09890	-0.95660	2.05330	C	5.65720	5.75270	5.26520
O	3.08450	-1.11390	3.35680	O	6.35980	3.50350	4.95130
O	0.65370	1.26100	10.78440	O	0.22760	-0.58510	8.97850
O	0.29060	3.24380	8.34400	O	-0.68510	1.20160	10.75500
H	0.08340	-2.30360	4.26120	H	3.22950	4.55290	4.38700
H	-2.17620	0.21650	6.94340	H	0.37000	3.89140	7.72710
O	-0.03550	-6.77310	1.97830	O	3.43080	6.60980	-0.09590
H	-1.76340	1.63570	11.45750	H	-2.85960	2.69980	10.40860
H	-0.33370	3.58900	10.96330	H	-1.39930	2.46950	7.73530
H	-1.53500	4.29160	9.94390	H	-2.23790	1.24600	8.63770
H	-2.62140	0.35830	9.42370	H	0.48820	4.98800	10.44450
H	-3.81980	1.10980	8.37380	H	-0.13820	3.76520	11.49150
H	-5.39930	1.77350	10.97180	H	-1.81730	6.40200	9.24570
H	-4.10650	0.64890	11.54460	H	-0.92260	5.34020	8.12310
H	1.21810	-0.54420	9.14030	H	1.52190	0.14430	6.89270
H	-1.34030	-0.34460	4.45760	H	2.30300	5.42670	6.41450
H	-0.21770	0.80480	3.76160	H	3.82870	5.52840	7.26230
H	-1.84660	1.86030	5.23390	H	1.88490	5.40700	8.77770
H	-0.19420	2.17940	5.74880	H	3.09290	4.17510	9.05670
H	1.03710	-2.92030	7.60430	H	2.00130	1.19710	4.42710

H	2.21850	-1.61990	7.38750	H	3.35980	0.86390	5.51200
H	1.44660	-3.72430	5.48170	H	3.26920	2.63450	3.13660
H	2.84240	-2.64570	5.46600	H	4.65640	1.92420	3.96150
H	-4.82120	3.12930	9.13940	H	-3.13730	4.93230	11.06670
H	-3.26780	2.91580	6.80630	H	1.50890	2.00870	11.48910
H	-1.64450	3.61720	6.67230	H	2.53790	1.81900	10.06950
H	-2.63250	4.03400	8.01630	H	2.43440	3.37390	10.90990
H	2.12600	0.95210	4.49040	H	5.38680	3.56070	7.40270
H	2.81820	-0.11930	5.71370	H	5.03470	2.03390	6.58770
H	1.76150	1.19150	6.19040	H	4.18500	2.46130	8.05660
H	-1.13830	-2.37790	7.27900	H	0.57680	2.80000	5.01710
H	2.67810	-3.78890	3.36400	H	5.32020	3.75000	2.41880
H	1.62650	-4.92280	1.44810	H	5.46250	5.89900	1.24050
H	0.74070	-3.49790	1.00100	H	5.50650	6.79680	2.72470
H	-0.68890	-3.64810	2.96080	H	3.18430	6.29440	3.22510
H	0.39230	-4.83320	3.71990	H	3.13910	5.02260	1.98860
H	-2.56100	-6.87420	2.82990	H	1.07780	7.71360	0.47310
H	-2.63280	-5.19870	3.41810	H	1.09610	7.33790	2.21010
H	-1.50710	-6.37090	4.13910	H	1.08500	6.03900	0.99530
H	-2.16850	-6.07190	0.48470	H	3.11330	9.15250	0.74110
H	-2.13690	-4.35690	0.94290	H	3.31180	8.78650	2.46720
H	-0.77260	-5.10010	0.07360	H	4.63040	8.49240	1.30770
H	3.61880	-3.86160	1.37030	H	7.25420	4.71620	1.84550
H	1.59620	-1.04880	1.08050	H	6.59890	6.20460	4.93250
H	0.05840	-1.27340	1.93240	H	4.86020	6.48960	5.13060
H	1.12560	0.11240	2.28030	H	5.79710	5.56980	6.33410
H	3.63160	-1.35440	2.57830	H	7.16700	3.73520	4.44230
H	0.96950	3.56850	8.96510	H	-0.73960	0.22670	10.65500
H	-0.58510	-7.49960	1.63570	H	3.07020	7.24000	-0.74420

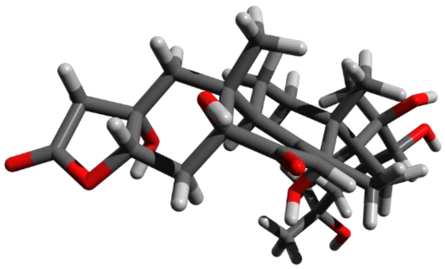

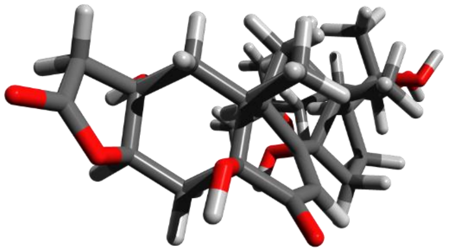

C	-1.38120	4.43350	9.71710	C	-4.56730	1.31610	8.76210
C	-2.27250	3.21250	9.63710	C	-3.92690	0.98320	10.13620
C	-1.65920	2.13380	8.75670	C	-2.38970	0.87690	10.12650
C	-0.32630	1.69500	9.40560	C	-1.71840	2.12990	9.49750
C	0.69060	2.87110	9.60470	C	-2.23680	2.32120	8.03280
C	-0.03430	4.07040	10.34130	C	-3.78880	2.49550	8.08420
C	-2.30150	5.37170	10.46760	C	-5.97290	1.81950	9.16990

C	-3.63350	4.96190	9.86880	C	-5.61110	2.40310	10.49280
O	-3.50960	3.75810	9.20220	O	-4.42050	1.97860	10.94870
C	0.29250	0.54190	8.61320	C	-0.20830	1.95740	9.43540
C	1.24150	0.84260	7.52600	C	0.37930	1.05080	8.43390
C	1.67260	2.09410	7.30190	C	-0.35400	0.59700	7.39410
C	1.29200	3.28960	8.19180	C	-1.79390	1.07740	7.13180
C	2.47020	2.45420	6.06830	C	0.16860	-0.49260	6.46270
C	3.72350	3.32270	6.38280	C	0.05720	-0.03710	4.97630
C	3.22250	4.62740	7.03600	C	-1.46470	0.21200	4.69830
C	2.42650	4.34910	8.32020	C	-2.02390	1.33210	5.60520
C	3.03650	1.36960	5.14850	C	1.62780	-0.94340	6.54910
C	4.07560	2.10860	4.28930	C	1.85930	-1.68760	5.21880
C	4.31570	3.50410	4.94120	C	0.69330	-1.27910	4.26010
O	-4.67090	5.59890	9.93680	O	-6.37530	3.06400	11.17680
O	-1.13560	5.01660	8.43110	O	-4.63470	0.21990	7.88580
C	1.83030	2.40550	10.56070	C	-1.63920	3.65300	7.47540
C	4.70830	2.59540	7.33330	C	0.88230	1.25560	4.66040
O	1.54740	3.21070	5.25670	O	-0.64200	-1.63350	6.67400
C	5.76740	4.02540	4.72120	C	1.11770	-1.26780	2.73740
C	6.15450	4.14410	3.20110	C	1.68060	-2.66270	2.24930
C	5.69810	5.38730	2.41720	C	0.69340	-3.83530	1.96910
C	4.18490	5.56380	2.27370	C	-0.15390	-4.35660	3.14280
C	3.76190	6.55610	1.17080	C	-0.88920	-5.69710	2.84680
C	2.23290	6.62900	1.10400	C	-1.67340	-6.13660	4.10000
C	4.34550	7.95360	1.38860	C	-1.88500	-5.54370	1.68240
O	7.60550	4.12990	3.14770	O	2.37450	-2.44780	1.04150
C	6.05520	5.34060	5.45370	C	-0.05320	-0.84170	1.82110
O	6.67880	3.05340	5.27820	O	2.16580	-0.32630	2.62630
O	-0.01710	-0.62870	8.84160	O	0.52130	2.54310	10.22130
O	-0.68180	1.15530	10.69770	O	-2.03740	3.26330	10.28050
H	3.64920	4.20600	4.43330	H	-0.07780	-2.04390	4.39860
H	0.50680	3.77790	7.60970	H	-2.38520	0.18090	7.37590
O	4.23480	6.05570	-0.08190	O	0.03620	-6.70070	2.50080
H	-2.46080	2.78670	10.63360	H	-4.34400	0.02550	10.52550
H	-1.52770	2.47950	7.72540	H	-2.02940	0.72550	11.16740
H	-2.35890	1.29000	8.69830	H	-2.10850	-0.03770	9.56630
H	0.61890	4.94940	10.35960	H	-4.21230	2.68630	7.07480
H	-0.20050	3.79150	11.39080	H	-3.97740	3.42300	8.67050
H	-2.32240	5.18430	11.54480	H	-6.38910	2.53250	8.42540
H	-2.10220	6.42330	10.24580	H	-6.69440	0.99780	9.36950
H	1.49730	-0.00210	6.89700	H	1.40150	0.72150	8.58210
H	2.59270	5.19670	6.34130	H	-2.04760	-0.71520	4.88690
H	4.05620	5.28710	7.29250	H	-1.65860	0.49980	3.65110
H	1.99770	5.29960	8.65880	H	-3.10780	1.46090	5.40270
H	3.14920	4.05820	9.08660	H	-1.52870	2.25980	5.27650
H	2.27500	0.91040	4.50830	H	1.91810	-1.52070	7.45280
H	3.52930	0.56880	5.71150	H	2.26500	-0.03550	6.54970
H	3.70200	2.23610	3.26670	H	1.82390	-2.79090	5.35190

H	4.98790	1.50610	4.22690	H	2.87220	-1.40760	4.85610
H	-1.95530	4.99170	7.90120	H	-5.10830	-0.51490	8.35550
H	1.43550	1.97430	11.48710	H	-1.87320	4.51000	8.14170
H	2.47310	1.65090	10.09710	H	-0.53590	3.59730	7.36620
H	2.46280	3.24180	10.87510	H	-2.07670	3.92560	6.49440
H	5.51760	3.26150	7.64830	H	0.70590	1.57710	3.61410
H	5.16630	1.71690	6.86850	H	1.97500	1.11600	4.76680
H	4.22220	2.23920	8.24640	H	0.61880	2.10300	5.32200
H	0.82370	2.61180	5.00620	H	-0.49250	-1.94520	7.60600
H	5.84440	3.23930	2.66760	H	2.41790	-3.00300	3.01210
H	6.15180	5.32730	1.41920	H	1.33020	-4.67970	1.62570
H	6.13870	6.28610	2.86540	H	0.02810	-3.55790	1.12590
H	3.76980	5.90870	3.22660	H	-0.93170	-3.61330	3.39590
H	3.74650	4.58410	2.04520	H	0.51970	-4.51540	4.01320
H	1.90620	7.28520	0.28910	H	-0.98620	-6.27720	4.96180
H	1.80600	7.00210	2.04070	H	-2.43100	-5.37260	4.37950
H	1.80420	5.64210	0.89520	H	-2.19470	-7.10030	3.91440
H	3.98840	8.64800	0.61920	H	-1.36470	-5.28600	0.73750
H	4.06960	8.35590	2.36860	H	-2.63520	-4.75470	1.90570
H	5.43730	7.94850	1.30450	H	-2.42170	-6.50100	1.50710
H	7.85260	4.10930	2.20520	H	3.15440	-3.06300	1.02590
H	7.04060	5.73590	5.18040	H	0.24910	-0.88640	0.75300
H	5.30720	6.10630	5.22850	H	-0.93460	-1.50120	1.96420
H	6.10200	5.19580	6.53650	H	-0.34170	0.20720	2.00710
H	7.53070	3.23250	4.82430	H	2.28540	-0.06930	1.67450
H	-0.85820	0.19960	10.55720	H	-1.75760	3.08790	11.21760
H	3.94260	6.66900	-0.77870	H	0.67030	-6.80870	3.25770

*Predicted couplings for each diastereoisomers of **1**.*

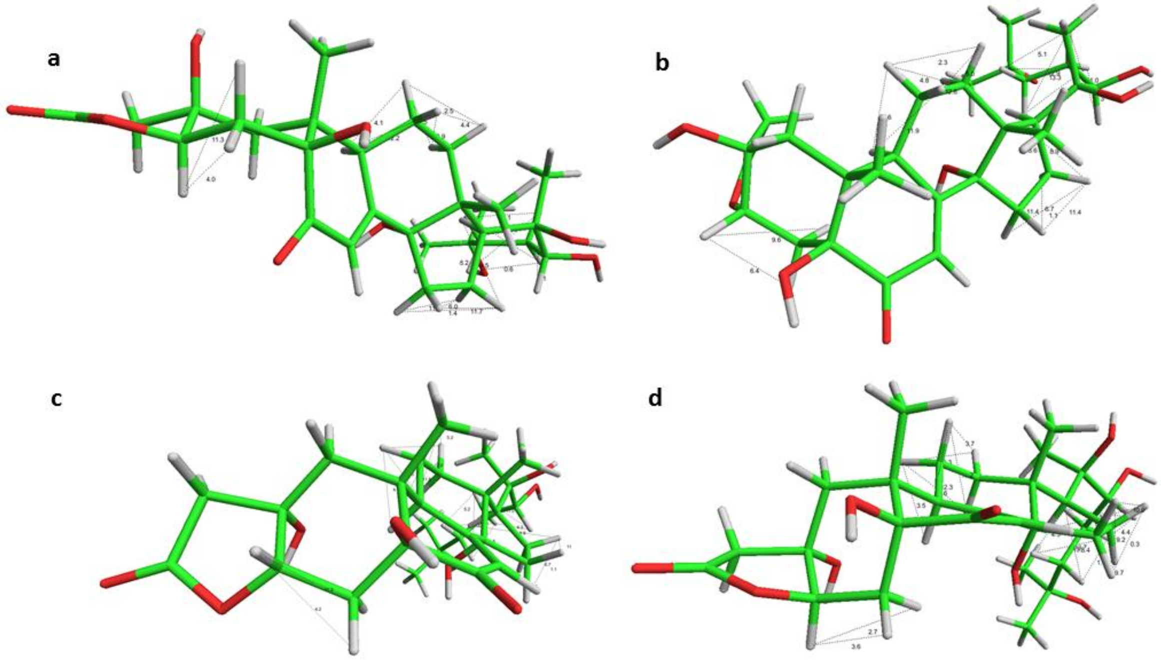
a
b
c
d
